# Oral stem cells in combination with hydrogels as biomimetic bioactive platforms for periodontal tissue engineering

**DOI:** 10.3389/froh.2025.1740392

**Published:** 2026-02-06

**Authors:** Noha Taymour, Meshari Alkandari, Mohammed Alkandari, Md Azizul Haque, Mohamed Ashraf El Arabi, Gamal Abdel Nasser Atia, Hany K. Shalaby, Omar Gamal, Dinesh Rokaya

**Affiliations:** 1 Department of Substitutive Dental Sciences, College of Dentistry, Imam Abdulrahman Bin Faisal University, Dammam, Saudi Arabia; 2 Department of Dentistry, Rumaithiya Polyclinic, Ministry of Health, Rumaithiya, Kuwait; 3 Department of Biotechnology, Yeungnam University, Gyeongsan, Republic of Korea; 4 Department of Orthodontics, Faculty of Dentistry, Suez Canal University, Ismailia, Egypt; 5 Department of Oral Medicine, Periodontology, and Diagnosis, Faculty of Dentistry, Suez Canal University, Ismailia, Egypt; 6 Department of Oral Medicine, Periodontology and Oral Diagnosis, Faculty of Dentistry, Suez University, Suez, Egypt; 7 Department of Oral Medicine, Periodontology, and Diagnosis, Faculty of Dentistry, Al-Azhar University, Cairo, Egypt; 8 Clinical Sciences Department, College of Dentistry, Ajman University, Ajman, United Arab Emirates; 9 Center of Medical and Bio-Allied Health Sciences Research, Ajman University, Ajman, United Arab Emirates.

**Keywords:** bone regeneration, engineering, hydrogels, oral stem cells, periodontal

## Abstract

Oral stem cells possess the capability to repair themselves and multipotent differentiation capacities, indicating that they have significant prospects in regenerative medicine. Nonetheless, due to the circulatory system's rapid clearance capability, they can only work consistently in certain areas for tissue healing. Thanks to their loose, porous architecture and high biocompatibility, hydrogels can act as transporters for oral stem cells, thereby delaying their release and enhancing their retention in specific regions. Oral stem cell-loaded hydrogels can be a valuable solution when specific areas require oral stem cells for optimal functioning, considering various types of hydrogels and the variables that affect their ability to transport and release oral stem cells. This review discusses the mechanistic processes underlying periodontitis, mentions current therapeutic techniques and their limitations, and explores oral stem cells and their regenerative capacities and design criteria of oral stem cells-laden hydrogels. Along with an assessment of the shortcomings in present investigations on the fundamental processes and innovative uses of oral stem cells in periodontal reconstruction, with the goal of offering fresh perspectives for upcoming research, the ongoing difficulties and disputes associated with oral stem cell-laden hydrogel personalized treatment options are also covered.

## 1 Introduction

Periodontitis is a chronic inflammatory condition of the tissues surrounding the teeth, resulting in breakdown of periodontal tissues and potentially leading to tooth extraction (1). Periodontitis' clinical signs include chronic gingival hemorrhage and tenderness, periodontal pocket formation, and bone loss (2). Over 11% of people worldwide suffer from periodontitis (3). Extensive periodontitis affects an estimated 800 million to 1.4 billion persons worldwide (4). Furthermore, periodontitis is intimately connected with the emergence of generalized inflammation, negative pregnancy results, and specific disorders (5–7). The negative change to the periodontal health impacts the quality of life of people (8).

Periodontal wound healing is a complicated process due to the periodontium's distinct morphology and makeup. By lowering the bacterial population in the periodontium and altering the surrounding milieu to minimize inflammatory processes, currently available traditional methods for periodontal therapy concentrate on halting the progression of periodontitis. To promote tissue reattachment, contemporary non-surgical procedures, like phase I therapy, and surgical treatments are employed to remove damaged tissue and thoroughly clean the root surface. By creating a lengthy junctional epithelial connection, these methods typically lead to a reconstruction process that heals the wound site. This non-physiological epithelial contact does not strongly connect the root surface and the surrounding gingiva (9, 10).

This technique cannot be regarded as real periodontium regeneration because it only partially repairs the injured cementum or alveolar bone. There are several restrictions on the treatment results, even if healing by repair may be useful in stopping further disease development and, consequently, any eventual extraction of teeth. First, the absence of tissue regeneration may restrict the therapeutic ability to improve the teeth's current movement. Furthermore, gingival recession is a common side effect associated with this therapy, which can make affected teeth more susceptible to root cavities, in addition to being aesthetically unappealing. Lastly, it is hypothesized that the region may be more vulnerable to future illness recurrence if the original morphology is not restored (11).

In recent decades, biotechnology advancements have created an opportunity to optimize periodontal regeneration (12). Oral stem cells possess self-renewal capacity while maintaining their “stemness” (13). Oral stem cells can differentiate in multiple directions, self-renew, and can regenerate into diverse tissues, organs, and cells (14).

There has been a great deal of clinical study on oral MSCs, and many important discoveries have been made (15–17). MSCs are extensively employed to repair several kinds of damaged tissues in addition to restoring hematological function and treating autoimmune illnesses (18). Based on distinct genomic profiling characteristics that influence clinical, pharmacological, and therapeutic options to the best possible disease treatment, personalized or targeted medicine is a rapidly developing area of healthcare (19–21). Scientists have discovered that combining oral stem cells with biomaterials can compensate for the shortcomings of oral stem cells in targeted reconstruction applications, thanks to advancements in biotechnology (22–24). Hydrogels are biomimetic platforms that have been widely employed in tissue restoration and repair (25). The application of hydrogels loaded with oral stem cells can enhance the survivability of these cells and facilitate their delivery to the defect site for prolonged *in situ* release, even though the hydrogels themselves are comparatively bioactive (26). Numerous investigations have demonstrated that hydrogels loaded with oral stem cells possess potentials in tissue regeneration and repair (27–31). In this article, we highlight the origins, capacities, extraction, and characterization techniques of oral stem cells, as well as a summary of their current-day utilization in periodontal regeneration. We then go over hydrogels and the parameters that influence the loading and release of oral stem cells. We present a summary of numerous methodologies for loading oral stem cells into hydrogels, as well as approaches to characterize hydrogels with oral stem cells. Moreover, we describe the implementation of oral stem cell-loaded hydrogels in regenerative periodontal applications.

## 2 Mechanisms of tissue destruction in periodontitis

Periodontitis is mostly caused by poor dental hygiene, as well as a variety of genetic and environmental factors. To avoid or treat periodontal defects, a thorough knowledge of these variables and the underlying molecular pathways is essential. Gingivitis is the first stage of periodontal inflammation, and the progression to periodontitis is influenced by a number of variables, notably the switch of aerobic bacteria in dental plaque to anaerobic ones, genetic changes, and host environment factors. Although microbes share in the etiology of periodontitis by directly compromising oral tissues, they can cause detrimental inflammation in the vulnerable host by forming extremely sticky biofilms on tooth surfaces.

**Figure 1:**
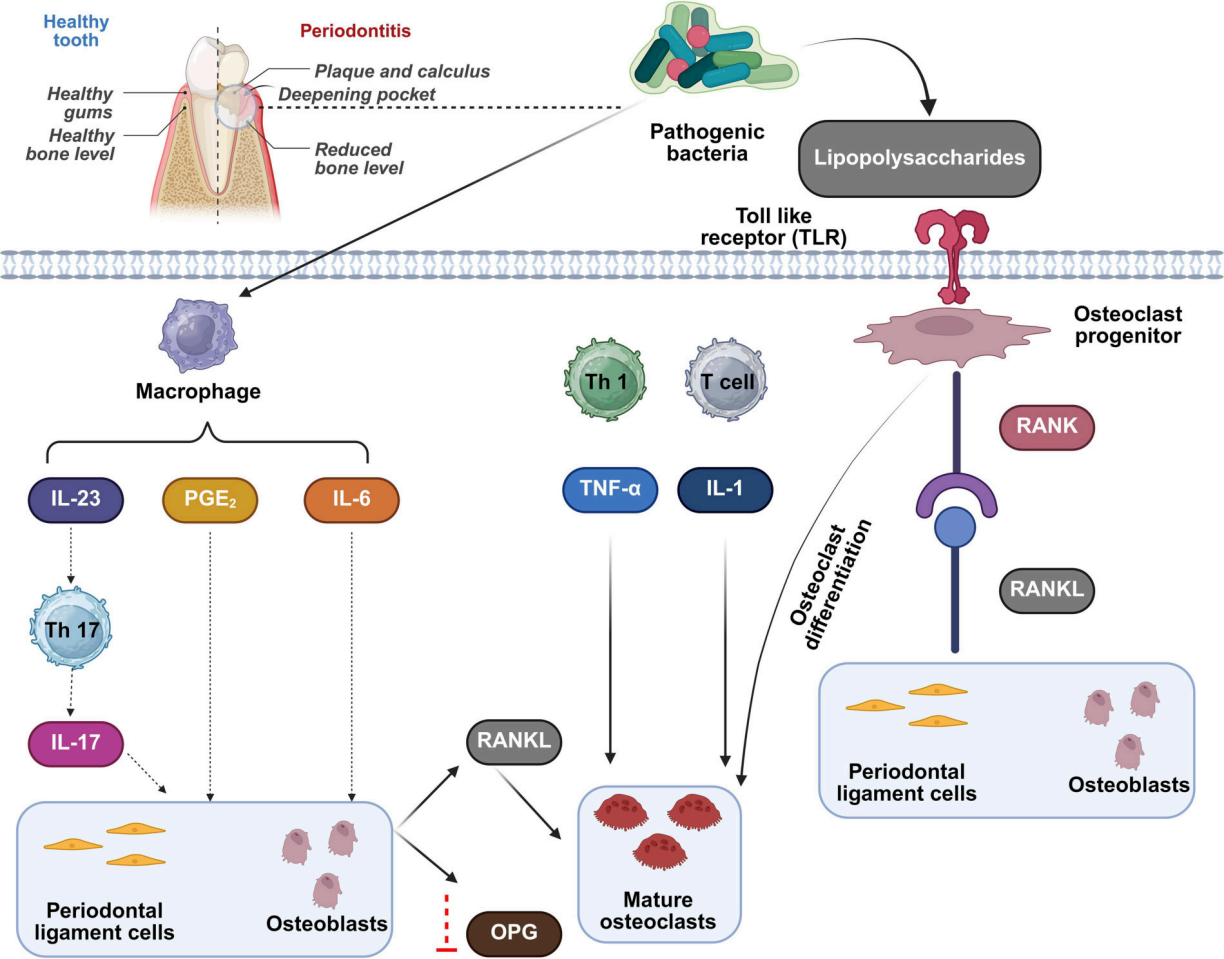
Mechanistic processes in periodontitis. This figure was created based on the tools provided by Biorender.com (accessed November 2, 2025).

Contemporary microbiological and mechanistic investigations have increased our comprehension of the microbe-human dynamics in periodontitis (Figure 1) (32–34). Furthermore, such research in people and experimental animals has demonstrated that (i) the periodontal dysbiosis is significantly more varied and complicated than formerly assumed, and (ii) the microbes implicated cause illness via polymicrobial synergies and dysbiosis (25, 35, 36). In other words; periodontitis does not occur due to just one or a limited number of bacterial species. Inflammation is a vital element of the overall biological process where host cells strive to confront numerous dangers such as invading infections, injured cells, and irritants (37, 38). The primary roles of inflammatory responses are to eliminate the starting point of illness or cell injury, to remove apoptotic and dead cells and contaminants, and to initiate tissue repair via adaptation of the local blood vessels and the release of several molecules interacting with neutrophils, along with various cell types (39, 40). In reaction to tissue infection, damage, or inflammatory conditions, neutrophils are the earliest cells to be recruited from the circulatory system to the diseased area (41, 42).

The mechanism of neutrophil extravasation involves a complex sequence of minimal- and high-affinity adhesion contacts between neutrophils and the endothelium (43). While neutrophils were formerly associated with acute inflammation, they are increasingly recognized as important actors in chronic inflammatory diseases (44). In fact, neutrophils are functionally flexible and perform previously unexpected roles, such as modulating adaptive immune leukocytes (45). Neutrophils, for example, can attract Th17 cells by producing the chemokines CCL2 and CCL20. Th17 are CD4+ T helper cells that produce interleukin-17 at inflammatory sites (46). In periodontitis, Th17 represents an osteoclastogenic fraction that connects activated T cells to pathological destruction of bone (47).

In addition, neutrophils secrete the B lymphocyte stimulant and a proliferation-inducing ligand (APRIL), two important cytokines that enhance the viability, division, and development of B lymphocytes into plasma cells (48, 49). Periodontitis includes both innate and adaptive immune components. To regulate periodontally associated immunological and inflammatory reactions, neutrophils, antigen-presenting cells, and T and B lymphocytes form an intricate series of collaborations with one another and with humoral pathways, such as complement (50). It is now well accepted that complement has activities beyond its conventional duty of marking and killing bacteria (51). Complement, for instance, can increase antigenic responses by engaging with Toll-like receptors on innate leukocytes, as well as modulate the activation and development of B cells and T-cell subtypes (52). In periodontitis, inflammation-driven bone loss is regulated by a trio of proteins, including RANKL, RANK, and osteoprotegerin. Stimulated T and B lymphocytes generate RANKL, and osteoblasts, in the inflammatory periodontium (53) The conjugation of cell-surface or soluble RANKL to RANK on osteoclast precursors causes osteoclast maturity and stimulation. However, the RANKL/RANK-mediated mechanism is inhibited by the decoy receptor osteoprotegerin (54). The optimal outcome of an inflammatory reaction is its prompt cessation, so that it does not develop into a chronic condition and have potentially negative consequences. In fact, ongoing inflammatory processes underlie numerous long-term conditions, particularly periodontitis (55).

## 3 Periodontal regeneration: challenges and opportunities

The continuous deterioration of dental anchoring structures, especially the PDL, is the hallmark of periodontitis, which can ultimately lead to tooth loss. Despite there are numerous therapeutic therapies available, most of them concentrate on symptomatic alleviation and do not provide strong evidence to back up the PDL's functional regeneration (56).

The cementum, PDL, and bundle bone make up the intricate organ known as the tooth attachment complex. From an embryological perspective, all of these structures have an ectodermal origin, which distinguishes them from bone tissues, which typically originate from the mesoderm (57, 58). The PDL fibers have been embedded into the cementum, a thin layer of mineralized tissue covering the dentin of the tooth root. The tooth cushioning mechanism is performed by the PDL. Sharpey's fibers, which are strings of collagen, make up its structure, in addition to veins and nerves that supply nutrition and sensation to the adjacent tissues (59, 60).

That portion of bone nearest the root surface is called the bundle bone. It differs from the alveolar bone due to the insertion of Sharpey's fibers. A bundle bone alone, without any alveolar bone, covers several teeth that are located outside of the alveolar bone housing. When attachment destruction happens, tissue enzymes break down the PDL and bundle bone, leaving the cementum on the tooth surface coated with calculus and bacterial plaque (61–63).

Oral stem cells have drawn a lot of interest as a possible pathway for PDL regeneration because of their affinity and MSCs characteristics. As a result, several therapeutic approaches have been created to boost the effectiveness of therapies centered around oral stem cells and achieve better clinical results. Because of their strong regeneration potential and immunity-modulating characteristics, oral stem cells have garnered considerable attention in bioengineering (64).

Oral stem cells are MSCs with higher multipotent differentiating potential, as was previously indicated. Relying on specific inductive circumstances, they can develop into odontoblasts, osteoblasts, etc. One approach that shows promise for correcting chronic dysbiosis is the use of homogenous MSCs *in situ*. With the potential for functional PDL repair, oral stem cell-based periodontal regenerative cell treatment has become a revolutionary method in periodontal reconstruction (65).

PDL is primarily made up of fibers, cells, and their neighboring extracellular matrix (ECM), with circulatory processes providing nutrients. About 50%–75% of the PDL fibers' volume is made up of collagen fibrils, mostly type I collagen, with lesser participation from types III, IV, V, VI, and XII collagen. The main fibers are the most important parts of the fiber bundles formed by these collagen fibers. By penetrating the alveolar bone on one aspect and the cementum on the other, these fibers serve as the tooth's anchorage. Furthermore, the major fibers incorporate elastic fibers (oxytalan), which control blood circulation and aid in the advancement of the neuronal and vascular system inside the PDL (66, 67).

The process of developing PDL is intricate and ever-changing. PDL fiber bundles are first formed when the tooth root grows, and then stem cells inside the dental follicle next to the root differentiate. These stem cells then develop into cementum and alveolar bone, where Sharpey's fibers and collagen fibers released by PDLCs mineralize to create a mature PDL. This system allows for continuous rebuilding and offers nutrition and mechanical support. Development between progenitors and PDLSCs may support tissue regeneration, maintenance, and repair at the cellular level. When subjected to mechanical stresses, osteocytes function as a crucial mechanosensory role (68).

Under mechanical stresses, osteoblasts and osteoclasts shape the alveolar bone by mediating bone production and breakdown. Certain mechanosensing non-coding RNAs, like microRNAs, may disrupt the synthesis of associated biomarkers. Additionally, factors that control bone and tissue metabolism activate several signal pathways. Under mechanical stresses, these elements aid in maintaining the normal state of the gingiva, PDL, and bone that make up periodontal health (69).

Biofilm and calculus are commonly removed mechanically utilizing ultrasonic equipment. Several evaluations have reported the advantages of local and systemic antimicrobial medications. Nevertheless, overconsumption of antimicrobial drugs may result in resistance to therapies and other adverse effects. The efficacy of periodontal treatment is determined by the clinician's knowledge and ability to eliminate periodontal pathogens. Phase I therapy is commonly utilized in conjunction with local or systemic antibacterial medicines. Inability to completely remove periodontal bacteria frequently results in a recurrence. Because existing therapies are ineffective and microbial resistance to available antimicrobial drugs has increased, there is a need to discover more efficient strategies and therapeutic approaches for treating periodontal bacteria (70).

Currently, numerous periodontal treatments can result in varying patterns and levels of periodontal reconstruction. Periodontal investigations have resulted in the development of some clinical procedures for high-level and more effective regeneration and restoration of periodontal defects, as well as enhanced implant site development (71, 72).

The application of biologics has ushered in a new era of tissue engineering. The progression of regeneration is thus greatly reliant upon the accessibility of suitable cell sources, stimulating and developmental components, and, in fact, the ECM released by these cells. While the precise events associated with periodontal healing are still unknown, suitable precursor cells have to move in the direction of the root surface and bond to it, where they can divide and differentiate into the components necessary for a functional attachment (73).

Furthermore, wound stability following surgical operations, defect filling with a 3D implantable device, and primary intention healing are essential issues that must be addressed to achieve true periodontal regeneration, according to existing research findings. Consequently, periodontal specialists will be able to efficiently control associated parameters to maximize clinical outcomes and enhance the reliability of periodontal regeneration treatments if they have a thorough understanding of the numerous elements that can impact clinical outcomes (74).

A novel way to enhance current periodontal disease treatment plans is through tissue engineering. The attraction of precursor cells capable of transforming into specialized regenerative cells, their division, and the formation of the unique components that comprise the periodontium are essential for the regenerative processes involved in periodontal wound repair. While utilizing the regenerative capacity of PDLSCs is an important tissue-engineering technique for periodontal regeneration, an additional strategy might include incorporating oral stem cells into a prefabricated 3D matrix that is subsequently placed into the defect following an appropriate *in vitro* culture. The origins of cells, improving engineering techniques, and Modifying biomaterials are all very difficult issues that call for novel approaches (75, 76).

The most fundamental dilemma regarding the comprehensive method to apply remains uncertain, notwithstanding opportunities for breakthroughs in biotechnology. to fundamentally alter how surgeons restore patients with periodontal abnormalities. For tissue-engineering treatments in periodontology, the available data are far from adequate, particularly for preclinical testing (77).

The majority of clinical assessments are restricted to treatments that demonstrate encouraging outcomes in these preliminary studies. There is little likelihood that tissue-engineering technologies will be widely adopted in clinical settings for periodontal regeneration in the near future (78).

It is evident that the adoption of regenerative approaches in periodontal treatment holds enormous promise for the future and can address a diverse set of patient requirements. To ensure that the advancement of innovative clinical therapies is backed by solid evidence and that such methods are effective, top-notch clinical trials of already accessible medications are always crucial.

There are several conventional therapies for periodontitis, each addressing a distinct part of the disease's origin and pathophysiology. Antibacterial medication therapy is often employed. Nevertheless, typical treatments become inefficient owing to medication resistance and the appearance of undesirable consequences.

Significant improvements concerning periodontal regeneration can be guided by a quick examination of results from current therapy modalities, underscoring the necessity of strong collaboration between fundamental research and clinical experts (79).

## 4 Overview of oral stem cells

**Figure 2:**
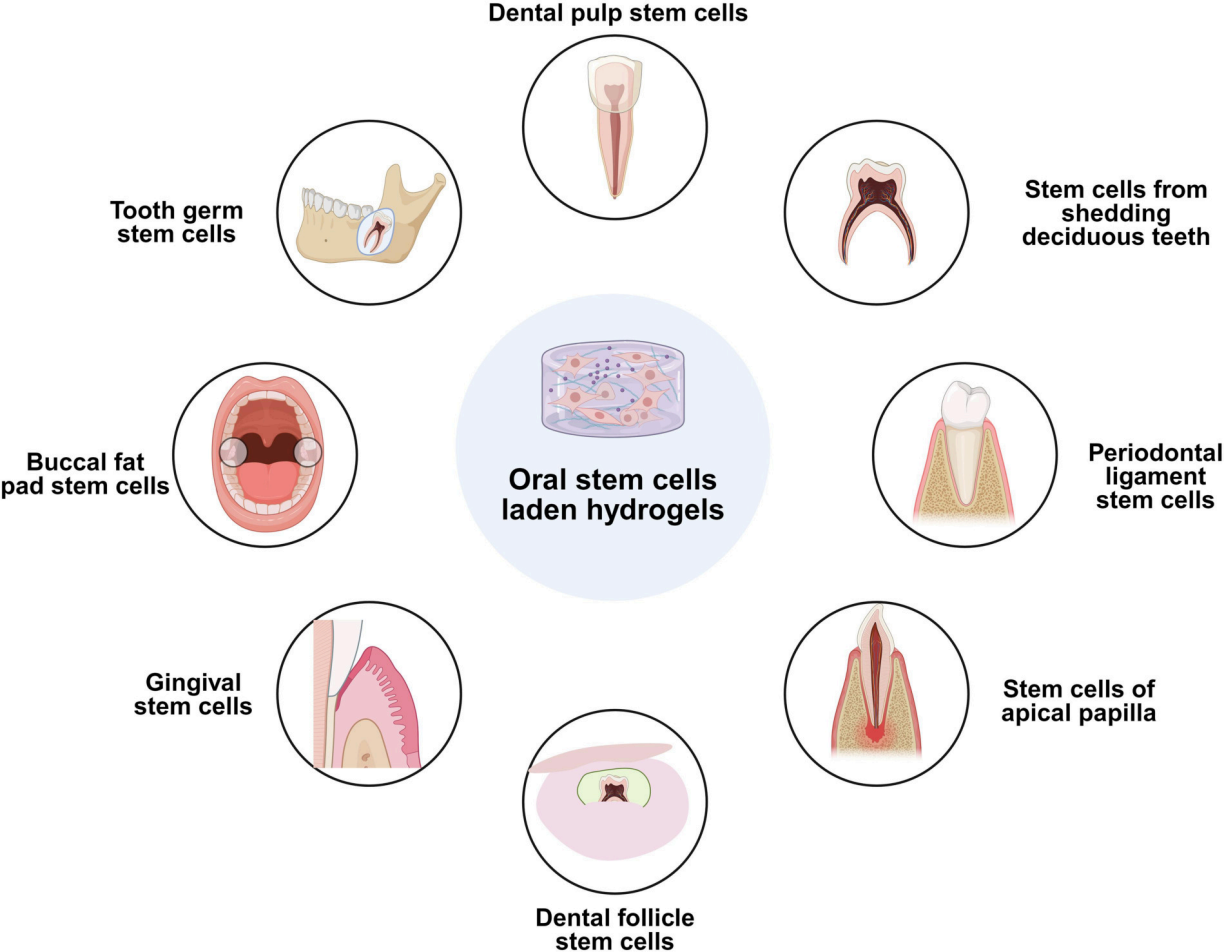
Different types of oral stem cells. This figure was created based on the tools provided by Biorender.com (accessed November 2, 2025).

Oral stem cells can adhere to plates and form colonies when grown in the right environments (Figure 2 and Table 1) (80). They are excellent options for tissue regeneration purposes because of their strong capacity to proliferate and propensity for multilineage transformation, encompassing osteogenic, chondrogenic, and adipogenic lines (81). Additionally, oral stem cells possess immune-modulating effects that enable them to regulate and preserve the periodontal microbiota's balance through the immune system's reaction (82). Additionally, injury signals can attract and deploy endogenous stem cells in particular niches to injured sites via a mechanism known as homing (83). They can transform into numerous kinds of cells at the site of damage, allowing for tissue regeneration (84).

**Table 1:** Oral stem cells uniqueness across various tissues (113, 130, 133, 138–142).

Dental stem cells	Source	Benefits	Drawbacks	Isolation time	Reference
DPSCs	Dental Pulp	Conveniently accessible and adequate source. Be isolated first, with further investigations and clinical applications. Strong ability to generate vessel-rich tissue. Increased differentiation capability of cementoblasts and osteoblasts	• Inadequate supply of viable autologous cells.	2000	(138)
SHEDs	Exfoliated deciduous teeth	More proliferative and potentially active than DPSCs. Capable of generating neural tissue. Maintains stemness following cryopreservation.	Insufficient source. Insufficient investigations and clinical uses.	2003	(133)
PDLSCs	Periodontal ligament	Increased proliferation potential. Stronger self-renewal capacity. Strong ability to construct cementum/PDL-like structures with functional attachment. Homogenous for functional regeneration. Reduced immunological antigenicity	Minimal access to healthy autologous cells. Decreased osteogenic ability.	2004	(139)
DFSCs	Dental follicle	Particular markers. Stronger proliferative potential. More flexibility and multipotential ability. Robust capacity for producing connective tissue and PDL-like tissue. Reduced immunogenicity	Restricted autologous cell source. Low cell count. Poor hard tissue formation potential. Insufficient research and clinical applications	2005	(143)
SCAPs	Apical papilla	Specialized markers. Increased proliferative potential. Greater flexibility and multipotential capability (derived from growing tissue). Better mineralization ability. Low immunogenicity.	Limited supply for autologous cells. Low cellular abundance. Lack of research and clinical applications	2006	(144)
GMSCs	Derived from healthy gingiva	Straightforward and sustainably accessible. Increased proliferative potential. Quick wound healing without scarring. Strong ability to create connective and bone-like tissues. Immunomodulatory and anti-inflammatory properties	Limited availability of healthy homologous cells. Insufficient cell count	2009	(141)
BFPSCs	Buccal fat pad	Ease of extraction. Low donor site morbidity. Multilineage capacities to develop into chondrocytes, osteoblasts, or adipocytes. BFPSCs exhibited fibroblast like shape.	In ability to promote PDL regeneration		(130)
TGSCs	Tooth germ	GSCs can develop into osteogenic, adipogenic, neurogenic, odontogenic, and chondrogenic lineages. TGSCs express considerably more SOX2, MYC, and KLF4 mRNAs than human embryonic stem cells.	Invasive approach. Questionable cellular viability. Demonstrate varied proliferating and developing characteristics.		

BFPSCs, Buccal Fat Pad Stem Cells; DPSCs, Dental pulp stem cells; SHEDs, Stem cells from human exfoliated deciduous teeth; PDLSCs, Periodontal ligament stem cells; DFSCs, Dental follicle stem cells; SCAPs, Stem cells from apical papilla; GMSCs, Gingival mesenchymal stem cells; PDL, Periodontal ligament; TGSCs, Tooth germ stem cells.

### 4.1 Dental pulp stem cells (DPSCs)

DPSCs are MSCs collected from the pulpal tissue of permanent teeth, often impacted wisdom teeth or teeth taken for orthodontic procedures. They are thought to be readily obtainable sources of MSCs, but they are associated with the significant drawback of compromising the tooth`s vitality in order to collect the pulpal tissues. These cells exhibit the classic MSCs features, including multi-potency, rapid proliferation, and immune-modulating activities (85). Moreover, DPSCs can develop into endothelial cells, as well as their angiogenic potential (86). DPSCs demonstrated mineralization capacity and osteogenesis (87). Due to their limited ability to generate cementum, the therapeutic value of DPSCs for PDL reconstruction can be considered doubtful (88).

DPSCs can be effectively isolated from both periodontally healthy (hDPSCs) and periodontally compromised teeth (pDPSCs). Both of them have no morphological variations in early passage cells. Cryopreservation can alter the shape of pDSPCs. Early passage cells exhibited no substantial change in the favorable transcription of MSCs' biomarkers CD73, CD90, and CD105. Nevertheless, repeated passaging and cryopreservation influenced biomarker transcription in pDPSCs. Both cell types show modest transcription of the hematopoietic b, such as CD34, CD45, and the MHC class II antigen HLA-DR. PDPSCs express more HLA-DR than hDPSCs. pDPSCs exhibit much slower growth rates and wound healing characteristics than hDPSCs. The migration capacity of pDPSCs can be significantly boosted during late passage following cryopreservation. There is no discernible change in osteogenic capability between them. Yet, pDPSCs have much poorer chondrogenic capacity than hDPSCs. However, pDPSCs demonstrated increased osteogenesis and chondrogenesis at late passage and following cryopreservation (89).

### 4.2 Stem cells from shedding deciduous teeth (SHEDs)

SHEDs offer an exceptional, non-invasive source of MSCs. Because of their ease of separation, multipotential differentiating capability, and low antigenicity, they may be a viable choice for periodontal regeneration. SHEDs are conveniently accessible via noninvasive techniques since they are extracted from deciduous exfoliated teeth (90). SHEDs have an elevated level of multiplication and immune-modulating capabilities, comparable to BMMSCs, which are more challenging to get. SHEDs, like DPSCs, are derived from dental pulp (17, 91). Yet, SHEDs express greater quantities of stemness-related genes than DPSCs, preserving greater flexibility during *in vitro* passaging (92). SHEDS are robustly proliferating and can transform into several cellular types, notably osteoblasts and adipocytes (17). SHEDs can produce functional vessel-like constructs following transplantation (93).

SHEDs, when maintained in osteogenic settings, dramatically enhance the pro-angiogenic activity (94). Recently, Kato et al. (95), verified SHEDS's pro-angiogenic function, which secretes angiogenesis-promoting molecules for primary endothelium cells (95). SHEDs exosomes-shuttled miR-222 promote aggregation and angiogenesis of PDLSCs via upregulation of TGF-β/SMAD system (96).

Gao et al. (97), found that multiple dosing of SHEDs lowered gingival hemorrhage, promoted new PDL attachment, and suppressed osteoclast development. Micro-computed tomography research revealed that SHEDs delivery substantially enhanced periodontal regeneration and alveolar bone mass. Additionally, a spike in the levels of CD206+ M2 macrophages was detected after the application of SHEDs (97).

### 4.3 Periodontal ligament stem cells (PDLSCs)

PDL comprises a range of cell types, notably MSCs called PDLSCs, that share in periodontal healing and reconstruction. Several investigations have been conducted to identify PDLSCs and examine their multipotency (98–101). PDLSCs are multipotent and can transform into osteogenic, neuronal, and adipogenic cell lines (102).

PDLSCs help maintain the physiologic homeostasis of periodontal tissues (103). PDLSCs have architectural and proliferative characteristics comparable to those of MSCs, as indicated by biomarkers (104). PDLSCs injections can improve periodontal regeneration, restore the decline in population diversity, and raise the number of colonies of *Bifidobacterium* and *Lactobacillus*. *In vitro*, PDLSCs prevent the development of periodontal pathogens, including *Staphylococcus aureus* and *Fusobacterium nucleatum*. The fundamental mechanism for activity is thought to entail the synthesis of LL-37 (105).

### 4.4 Stem cells from apical papilla (SCAPs)

SCAPs are a distinct population of MSCs found in the apical papilla of young permanent teeth. These cells have critical MSCs behaviors such as particular biomarker activity, self-renewal, division, mobility, multipotency, and immunosuppression features (106). Furthermore, significant evidence suggests that SCAPs can develop into several types of cells, like osteoblasts and odontoblasts, which may be a promising option for periodontal engineering (106, 107). Clinical evaluations, CT scans, and histopathological data revealed that SCAPs might dramatically increase periodontal regeneration 12 weeks following injection into a periodontitis animal model (108). This work validates the idea of employing SCAPs as an appropriate substitute stem cell resource for PDL regeneration in the future. Moreover, under inflammatory settings, the human apical papilla was shown to be mildly inflamed, retaining SCAPs viability and stemness while increasing its osteogenic and angiogenic capabilities (109).

### 4.5 Dental follicle stem cells (DFSCs)

DFPCs are a kind of dental MSCs that live in the dental follicle and is essential for tooth formation and function. DFPCs are neural crest-derived cells with multipotential transformation capabilities. More significantly, they offer advantages over other stem cells, such as ease of isolation and abundance, dynamic self-regenerating capacity, and absence of ethical concerns, which makes them an appealing choice in biotechnology. DFSCs are MSCs located in the tooth follicle and so share biological similarities with PDLSCs (24). In the inflammatory periodontal milieu, DFSCs have the potential to stimulate the division, osteogenic, and adipogenic transformation of both PDLSCs and inflammation-prone PDLSCs to various extents. Furthermore, when cultured together with DFSCs, the cell layering and ECM of PDLSCs/inflamed PDLSCs sheets expanded *in vitro*, whereas periodontal regeneration accelerated *in vivo* (110).

DFSCs, which are more undeveloped and demonstrate more DSPP than PDLSCs, can produce periodontal ligament (PDL) like constructions *in vitro* (111). A range of pluripotency biomarkers, notably octamer-binding transcription factor 4 (OCT-4), and NANOG, were demonstrated to be produced by DFPCs, confirming their multipotency and self-renewing capabilities (112). In contrast to other oral MSCs, DFSCs have a greater proliferation ability and osteogenic characteristics (113). Following *in vivo* implantation, DFSCs can replace the root by generating cementum and PDL (114). DFSCs had greater concentrations of osteogenic biomarkers, such as RUNX2 and ALP, than DPSCs and SHEDs (115).

### 4.6 Gingival mesenchymal stem cells (GMSCs)

GMSCs are a separate homogeneous group of MSCs that arise from neural ectomesenchymal tissues (116). GMSCs distinguish themselves from other oral MSCs due to their simplified accessibility and availability, as well as their exceptionally lengthy cultivation sustainability, lack of tumorigenicity, and persistent telomerase activity (117, 118).

Mitrano et al. (119), identified and analyzed GMSCs, which meet the basic specifications for MSCs, including multilineage differentiation, expression of MSCs markers, and increased adhesion (119). GMSCs demonstrated immune-modulating properties similar to those of other oral MSCs, inducing anti-inflammatory macrophage polarization and inhibiting osteoclasts, thereby lowering periodontal bone resorption *in vivo* (120). GMSCs' osteogenic ability has been established, and when transplanted into rats' gingival lesions, they restored healthy tissue (121). In rats, GMSCs-derived CM had a similar potential to stimulate PDL regeneration as PDLSCa-derived CM (122). *In vivo* transplantation of GMSCs can successfully regenerate bones (123).

### 4.7 Buccal fat bad stem cells (BFPSCs)

The mouth houses a unique fatty tissue known as the buccal pad of fat or Bichat's pads (124).

Several investigations have employed BFPs as an autogenous transplant to reconstruct small- to medium-sized maxillofacial lesions (125–127). Furthermore, they are currently being utilized to generate MSCs called BFPSCs, which share characteristics and behavior with the more well-known dermal MSCs (128). This innovative procedure for producing BFPSCs has significant advantages because BFP collection is simple, involves just a small incision with local anesthetic, and generates low donor-area complications (129).

Farre-Guasch and colleagues (130) were among the initial researchers to identify BFPSCs (130). Utilizing rhBMP-2, Hiraishi et al. (131), verified the osteogenic capacity of BFPSCs. Additionally, only in cells containing recombinant bone morphogenic protein-2 (rhBMP-2) and osteoinductive reagents (OSR) were adipogenic genes readily visible. Yet, transplanting BFPSCs grown in this setting resulted in the most significant *in vivo* bone production. Therefore, when subjected to rhBMP-2 to induce mature osteoblastic development, BFPSCs consistently produced manufactured bone (131).

In surgically produced defects in rabbits' jaws, BFPSCs and cellular matrix (CM) both promote bone regeneration, indicating that BFPSCs primarily enhance bone regeneration via releasing paracrine substances. Regenerative dentistry is significantly influenced by the findings of MSCs' paracrine action on bone regeneration, and utilizing their CM can help tackle several problems and issues associated with cell transplantation. Specifically, CM provides greater convenience for medical professionals during clinical applications and is portable and easy to store (132). It is predicted that buccal fat pad tissue could offer valuable transplant material because it is readily accessible and has a rich vascularized area; however, further research is necessary to confirm this.

### 4.8 Tooth germ stem cells (TGSCs)

TGSCs have grown in popularity as a cell origin with great promise for transformation into many lineages. MSCs with endothelium and epithelial cells are essential for tooth formation, rendering them an ideal cellular reservoir for dental regeneration. TGSCs were discovered by a pedodontist, Dr. Songtao Shi, while working on his six-year-old daughter's deciduous teeth in 2003 (133). The tooth germ is a cluster of primitive cells that participate in the formation of teeth and related structures (134). MSCs-like properties can be observed in cells generated from the third molar tooth germ. MSCs-specific surface antigens are expressed by human dental germ cells (135). TGSCs' multipotency enables them to develop into osteoblasts, odontoblasts, adipocytes, and brain cells. Human TGSCs exhibit immune-regulatory characteristics (136). Guzman et al. (137), demonstrated that the application of human TGSCs has immune-suppressing actions in mice (137).

## 5 Creating biohybrid platforms utilizing cell-laden hydrogels

Regenerative dentistry has an increasingly important role in therapeutic therapy. Under some conditions, oral stem cells can differentiate into several lineages. Scientists are particularly interested in utilizing them in bioengineering. To optimize the effectiveness of MSCs, material research can offer biomimetic platforms as well as reliable methodologies for understanding the numerous differentiation processes involved in MSCs development. Pharmaceutical applications are portrayed in biomaterials development via macromolecule-inspired hydrogels. Hydrogels possess numerous properties due to their unique composition (145). First, hydrogels possess an inherent softness that can be adjusted by modifying the level of cross-linkers, allowing the matrix to exhibit optimal elasticity and strength under various conditions (146). Secondly, hydrogels are safe to utilize with living things because they are biocompatible. Hydrogels should have minimal impacts on cell survival, according to *in vitro* testing on hydrogel cytotoxicity (147). Moreover, they should act as supportive scaffolds for cell attachment, division, and transformation, and promote mass transfer, which is another source of hydrogels' biocompatibility (148).

### 5.1 2D design VS 3D design

Extracellular matrix (ECM) components, including proteins and glycans, are secreted by living cells in tissues to form complex networks that regulate cell behavior and enable cells to fulfill specific roles, providing crucial cues and substances for cell migration and proliferation (149). Nevertheless, since 2D well plates are unable to facilitate biomacromolecule agglomeration or the spatial space necessary for cell adhesion, they are absent from conventional 2D cell culture techniques (150). Complex photophilic polymeric chains, protostructures, and elevated water levels make hydrogel matrices the ideal substrates for simulating *in vivo* cell culture conditions (151). As a result, hydrogels are frequently employed as synthetic substrates or frameworks in biohybrid networks, offering the benefits of long-term survivability, self-healing, and bottom-to-top construction (152).

Cells can be directly embedded in the hydrogel matrix, seeded on films or fibers, or seeded on a decellularized matrix to create common hydrogel-based biohybrid systems (153). The development of tissue engineering has led to notable breakthroughs in sophisticated manufacturing techniques, with 3D bioprinting being the most promising of these (154–157). Hierarchical designs can be formed from bottom to top thanks to this technological capacity to build cells at the microscale in customizable 3D areas (158). 3D printing is more reliable than traditional manufacturing techniques when it comes to creating various biological networks and systems, and accurately specifying the architecture of cells. These benefits over conventional manufacturing techniques have been demonstrated in domains such as clinical healthcare, biological science, and organ regeneration (159). Inkjet, extrusion, and photosensitive approaches are among the various types of 3D printing techniques that are primarily accomplished by sequential layering of sensitive inks (160, 161). These techniques are also well-suited for cell-laden, sensitive hydrogel engineering, which enables a variety of biohybrid functions. It is now simpler to load cells into scaffolds to assist cell functionalization, thanks to the 3D printing process that creates hydrogel scaffolds (162). There are several advantages to using hydrogels as platforms for cell seeding, particularly in regenerative applications, such as bone regeneration and repair. As cells are suspended directly in the hydrogel solution, cell-containing hydrogel bioinks enable the direct fabrication of 3D designs (163).

Because of its intricacy, reconstruction of the periodontium requires the synchronized repair of several structures. Numerous 3D printing processes, such as the freeform reversible embedding of suspended hydrogels (FRESH), employed by Lin et al. (164) are utilized to carry out this operation (164) with a bioink incorporating type I collagen, thereby building collagen microfibers. The findings demonstrated that the growth, attachment, and vitality of the cytoskeleton were satisfactory, and the PDLSCs were effectively implanted (164).

**Figure 3:**
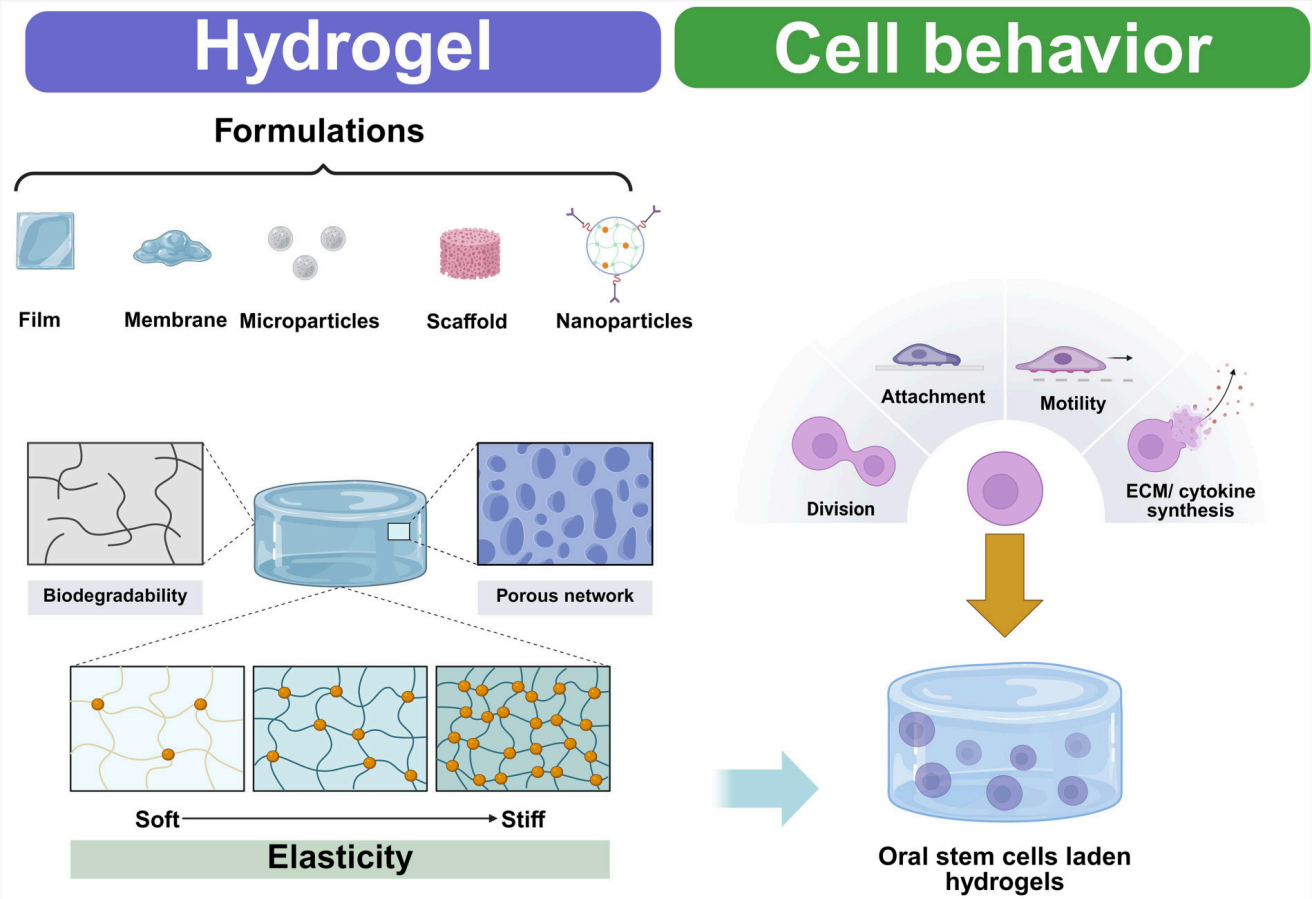
Design criteria of oral stem cells-laden hydrogels for regenerative purposes. This figure was created based on the tools provided by Biorender.com (accessed November 2, 2025).

However, Tian et al. (165) utilized a hydrogel, hydroxyapatite nanoparticles, and PDLSCs to combine synthetic and natural materials, thereby generating a bioink (165). This bioscaffold enhanced the mechanical characteristics and swelling capacity while also effectively stimulating cellular viability, division, and differentiation. Zhu et al. (166),, also employed PDLSCs in GelMA hydrogel at various levels (3%, 5%, and 10%). The addition of PDLSCs helped create new cells, but the 10% GelMa demonstrated lower cell longevity (166). One benefit of hydrogels is that they may be administered directly via injection into the targeted region using less invasive techniques (Figure 3). Kandalam et al. (167) effectively enclosed GMSCs in PuraMatrix™, a self-assembling hydrogel. The GMSCs immobilized with 0.5% PuraMatrix showed exceptional attachment and multiplication ratios.

### 5.2 Cost-effectiveness and manufacturing of hydrogels

Hydrogels must be both economical and simple to manufacture, in addition to having a therapeutic impact, to be commercially successful and widely used. Enhancing the ability to attract native cells might encourage the therapeutic application of biomaterials by eliminating the challenges and costs related to the development, preservation and delivery of cellular materials, besides safety and ethical issues (168).

Hydrogels can be manufactured from a broad spectrum of materials, including both artificial and organic sources, and can be utilized in various formulations (169, 170). Natural hydrogels exhibit good biological compatibility and minimal immunological response (171, 172), yet this is often accompanied by inadequate scalability and limited control over mechanical properties (170, 173–175).

Manufactured hydrogels possess an identifiable framework, consistent material sources, and an extended shelf life, and can be constructed in several batches with reproducibility. The fundamental challenges are their low bioactivity, antigenic byproducts, and static makeup, which provides no biological data to cells. Thus, they may be employed in combination with bioactive molecules to mitigate these shortcomings (176–178).

Isaac et al. (179), used immersed electrosprayed polyethylene glycol (PEG) hydrogel microparticles, which were subsequently used to make tetrazine click chemistry to prepare microporous annealed particle-based hydrogels (TzMAP) by attaching norbornene-loaded PEG hydrogel microspheres. Incorporating PDLSCs during annealing resulted in cellular viability proportions of 87% ± 5% at 24 h. The inclusion of PDLSCs and platelet-derived growth factor (PDGF)-BB into TzMAP led to enhanced cellular division and movement, which are crucial for periodontal tissue regeneration (179).

Hydrogels can be carefully manufactured for optimized cell dispersion and tissue formation through self-assembling or 3D printing processes (180–182). For encapsulation of PDLSCs, a bioprinting technique used injectable composite hydrogels made of GelMA and PEG dimethacrylate (PEGDA). The use of PEG improved droplet control. *In vivo* tests demonstrated that PDLSCs-containing hydrogels stimulated bone formation in rat periodontal defects when compared to hydrogels lacking cells (183).

### 5.3 Mechanical properties

Natural polymeric hydrogels derived from microbes, plants, or animals have garnered considerable interest recently, owing to their exceptional biodegradability and biocompatibility (184). However, their practical uses are limited by their weak mechanical characteristics, unpredictable rates of breakdown, and the likelihood of immunological responses. To meet the potential needs for dental applications, both artificially produced and organic composite hydrogels have been proposed. The most often used injectable matrix for cellular transportation is composite hydrogels (185).

Tissue engineering is based on hydrogels, which provide a framework for cell adhesion, proliferation, and transformation. By modifying their functional components, cross-linking techniques, and synthetic materials, the physical-chemical characteristics of hydrogels can be tuned to satisfy the unique biomechanical requirements of various tissues. Because hydrogels can be customized for specific medical applications, they have been utilized in medication delivery, regenerative medicine, and other fields of medical research. Many novel hydrogels have been manufactured to enhance the interaction between targeted cells and hydrogels, thereby promoting tissue regeneration (186).

Rapidly cured microporous hydrogels based on gelatin and GelMA were shown in a prior work (187). Via photopolymerization and enzymatic cross-linking, these hydrogels set after 2.5 min of injection, enabling consistent cell dispersion and substantial cellular dissemination and division within a week. Furthermore, these hydrogels may carry hMSCs primed with interferon-gamma, increasing the production of anti-inflammatory substances, including interleukin-6 and prostaglandin E2. Therefore, these hydrogels offer a potential method of delivering cells. Another safe method for cell transplantation is the use of hydrogel particles. Hydrogel particles, which are formed of microscopic 3D network structures built of biopolymers, shield cells from shear pressures during transplanting, maintaining cell viability (187).

According to recent research, the physical characteristics of hydrogels (like the stiffness, durability, surface charge, and other physical factors of ECM can be altered to control cell expansion, division, and transformation; therefore, they impact the regenerative processes. The majority of research has produced hydrogels with varying stiffness by altering their composition or applying external stimuli. The investigation of variations in cell activity has also made extensive use of these hydrogels with varying stiffness. For instance, the stiffer the hydrogel, the more likely the oral stem cells can develop into bone, making it a potential option for regenerative dentistry (188, 189).

He et al. (190) developed and evaluated stiff transglutaminase-crosslinked gelatins (TG-GELs) with SDF-1α and/or IL-4 as a potential platform in periodontal therapy. According to their findings, IL-4 could encourage the transformation of Mφs to the M2 phenotype, which may promote BMSCs' osteogenesis *in vitro*. Furthermore, the inclusion of SDF-1α might continuously encourage the functionality of BMSCs. Periodontal regeneration was considerably enhanced after implantation of these bioactive gels into periodontal defects as opposed to TG-GELs carrying either IL-4 or SDF-1α alone. These findings suggest that improving the ability of high-stiffness hydrogels to modulate macrophages (Mφ) and attract cells is a feasible and successful method for performing targeted periodontal regeneration. Therefore, if the synchronized crosstalk between stem cells and Mφs is appropriately guided, very simple, designed biomaterials can give significant functional advantages and good regeneration results with the help of carefully chosen signaling molecules (190).

### 5.4 Biodegradability

Biodegradability is a key design factor that influences the pharmacokinetics and pharmacodynamics of hydrogels. Hydrolysis of ester, carbonate, or anhydride chains; metabolic breakdown of peptide, polysaccharide, or proteolysis by enzymes like matrix metalloproteinases; or redox- and pH-triggered disintegration of dynamic covalent bonds. To optimize decomposition dynamics, polymer molecular weight, crosslinking capacity, hydrophilic properties, and the introduction of labile functional chains must all be balanced out. Customized breakdown allows synchronicity with tissue healing timeframes, ranging from fast disintegration for bursting delivery to delayed degradability for extended reinforcement. Furthermore, breakdown metabolites must be non-toxic, immune-compatible, and quickly eliminated to avoid long-term inflammation (191).

### 5.5 Biocompatibility

Biological compatibility is determined by the hydrogels' capacity to keep encapsulated or invading cells viable and functioning. Polymeric purity, minimal endotoxin concentration, the absence of harmful crosslinking by-products, and the preservation of homeostasis during gelation are all important variables. Moderate gel formation settings are required, whether by light triggering and enzymatic processes. Mechanical qualities also impact cellular activity; hydrogels with insufficient durability can impede cell dissemination, relocation, lineage determination, and ECM deposition. To avoid hypoxic or apoptotic areas inside cell-laden structures, nutrition, oxygen, and waste byproducts flow must be optimal (192).

Biocompatibility refers to the capacity of hydrogels to interact with host tissues without causing unfavorable immunologic or inflammatory responses. After administration, hydrogels interact with immune cell populations, notably macrophages. Biomaterial chemical composition, charge concentration, crosslinking methods, architectural characteristics, and decomposition products all impact the behavior of macrophages toward pro-inflammatory or pro-regenerative phenotypes (190).

### 5.6 Sensitivity to environmental factors

Stimuli-sensitive hydrogels provide dynamic features that allow for sensing and responding to surrounding signals. They can be activated by heat, light, pH, and chemicals, thereby imparting them with environment-sensitive properties (193–195). Along with these qualities, hydrogels also exhibit other common traits, such as softness, swelling capacity, and water retention capacity. Additionally, the polymer chains in hydrogels can be purposefully altered or tailored to enhance particular qualities (196). For instance, hydrogels can acquire stimuli-sensing capabilities by linking polymers to biological molecules, which adds living things, such as proteins, to polymer chains (197). Temperature-sensitive platforms encounter sol-gel transformations around physiological temperatures when the hydrophobic-hydrophilic balance shifts within the polymeric matrix. pH-sensitive hydrogels with ionized motifs exhibit swelling or deswelling activity depending on protonation status. Enzyme-sensitive hydrogels include peptide chains or degradable components that can be preferentially degraded by illness or tissue-related enzymes. Glucose and redox-reactive hydrogels use reactive boronate ester bonds or oxidative breakdown of highly reactive links to provide tailored medication release under metabolic or inflammatory conditions. Light-sensitive hydrogels contain photosensitive molecules, allowing for precise time- and space-based manipulation of gelation and breakdown. These behavioral modifications improve accuracy, decrease undesirable outcomes, and increase therapeutic variety (198).

### 5.7 Immune-modulating properties

Hydrogels intended for periodontal regeneration should reduce acute inflammation, prevent persistent fibrotic encapsulation, and optimally establish an immunological microenvironment favorable to tissue regeneration. The inclusion of immune-modulation elements, bioactive substances, or anti-inflammatory drugs might help modulate immunological reactions at the location of administration. Additionally, by influencing redox reactions, hydrogels can be utilized for tissue restoration (199). Elevated ROS synthesis can control the associated biological reactions during tissue regeneration and raise the amount of MSCs-related protein secretome (200). Additionally, some composite hydrogels can alter the behavior of macrophages by making the hydrogel more rigid, changing them to an anti-inflammatory M2 type, and encouraging tissue reconstruction by controlling associated immunological and inflammatory responses (201). By altering the associated biophysical characteristics of hydrogels and influencing the presentation of relevant factors, it can generally impact the phenotypic or proliferative maturation of cells.

Despite the use of modern treatments, chronic oral inflammatory illnesses such as pulpitis, periodontitis, and peri-implantitis present serious clinical problems and frequently cause irreparable tissue loss. By actively encouraging the suppression of inflammation and tissue regeneration while maintaining host defense, specialized pro-resolving mediators (SPMs) provide a revolutionary treatment paradigm. Nevertheless, SPMs' low affinity to inflammatory tissues, limited bioavailability, and quick disintegration impede their practical application. Promising platforms to overcome these obstacles include smart biomaterial-based delivery systems, particularly stimuli-responsive hydrogels. These devices enhance the stability and therapeutic efficacy of SPMs by enabling their regulated, localized, and environmentally induced release (202).

Hydrogel-mediated SPM administration not only reduces inflammation but also maintains tissue integrity and encourages regeneration, according to preclinical research in oral inflammation models. To facilitate clinical acceptance, future strategies will focus on enhancing dosage procedures, ensuring long-term bioactivity, and addressing manufacturing and regulatory challenges. Biomaterial-based approaches have an opportunity for transforming the medical management of oral inflammatory illnesses and promoting regenerative dental treatments by improving the delivery and prolonged bioactivity of SPMs (203).

Numerous inflammatory conditions, such as periodontitis, are characterized by excessive ECM degradation by MMP-1. Under optimal circumstances, MMP activity is carefully regulated—for example, by tissue inhibitors of metalloproteinases (TIMPs)—to maintain homeostasis. MMPs hydrolyze peptide bonds with a high degree of amino acid affinity. Nevertheless, MMP activity persists under pathophysiological conditions, leading to unfavorable alterations in tissue structure and function, and accelerating the course of the disease. Over the past 25 years, numerous investigations have been conducted on the construction and creation of molecules that block MMP activity in an effort to alleviate this (204–206).

Huang et al. (207) constructed an injectable dual-crosslinked protein hydrogel by integrating gelatin and bovine serum albumin (BSA) using a rapid, straightforward, and cross-linker-free manufacturing technique. Its cross-linked, interconnected design offered extra support to optimize thermal stability and mechanical characteristics. CD/BSA/GEL hydrogel was produced by adding the potent oxidant ClO_2_. To administer targeted high-concentration medication effectively, this hydrogel is easily injectable into periodontal pockets. It reacted with and alleviated elevated protease components in the periodontal inflammatory milieu, allowing for the prolonged release of ClO_2_ and bioactive cargo. This system downregulates the expression of inflammatory genes and maintains cell motility, expansion, division, and osteogenic potential while exhibiting substantial antimicrobial characteristics. The CD/BSA/GEL hydrogel successfully minimized inflammatory reactions and encouraged periodontal bone regeneration (207).

The prevalence and progression of periodontitis are clearly positively correlated with H2S, a common metabolite of periodontal bacteria. H2S can control numerous biological processes at physiological quantities. Nonetheless, excessive H2S in the periodontal pocket can exacerbate the progression of periodontitis by inducing oxidative stress, releasing proinflammatory cytokines, causing mitochondrial damage, and promoting apoptosis in human gingival fibroblasts. Even worse, by preserving bacterial redox balance and boosting antibiotic resistance, H2S promotes bacterial survival and growth. However, eliminating H2S is often overlooked when treating periodontitis. To improve the management of periodontitis, Xie et al. (208) developed a type of hyaluronic acid methacryloyl/ZnO (HMZ) hydrogel with the ability to scavenge H2S. By reacting with ZnO, the HMZ hydrogel was able to eliminate H2S and had high injectability and cytocompatibility. Consequently, the HMZ hydrogel restored mitochondrial homeostasis, reduced inflammation mediated by the cGAS-STING signaling pathway, and increased cell survival from 13% to 120% for human gingival fibroblasts and from 22% to 94% for human periodontal ligament fibroblasts after 48 h. Additionally, the HMZ hydrogel demonstrated effective plaque biofilm removal and adequate antibacterial properties *in vitro* and *in vivo*. In summary, a potential approach based on H2S elimination was created to increase the efficacy of periodontitis treatment (208).

### 5.8 Organization of cellular activities

Since hydrogel stiffness affects stem cell activity, including development, attachment, and relocation, it is an essential component of stem cell administration (209). To influence the destinies of stem cells, investigators can modify the stiffness of hydrogels. For instance, stiffer hydrogels could stimulate osteogenic development, whereas softer hydrogels may support adipogenic transformation or preserve stem cell properties. Due to this adaptability, specific niches may be created that can enhance the effectiveness of stem cell treatments (210, 211).

Osteogenic differentiation results from a greater nuclear-cytoplasmic ratio of Yes-associated protein (YAP)/transcriptional coactivator with PDZ-binding motif (TAZ) on the surface of the stiffer hydrogel. The nuclear-cytoplasmic proportion of YAP/TAZ is lower on the scaffold's less rigid surface, causing the cells to develop into adipocytes (212, 213).

Since they may be utilized to carry medications and cells and replicate the chemical makeup of the extracellular matrix, hydrogels are regarded as promising biomaterials. Properly controlled synthetic procedures can produce artificial polymeric hydrogels with well-specified, well-defined chemical compositions, precise molecular weights, enhanced stiffness, and customizable microstructures. Nevertheless, synthetic polymer hydrogels are often either biodegradable or biocompatible, which makes them inappropriate for cell reinforcement (21).

Although hydrogels have demonstrated potential as transporters for stem cell transplants, there are still obstacles (19). Cell mortality after delivery, limited cell attachment after transplantation, challenges to extended cell longevity, and insufficient reinforcement are all significant factors influencing the effectiveness of periodontal regenerative therapies (204, 214). Currently, there is no ideal hydrogel cellular vehicle to address these challenges (215, 216).

## 6 Integration of oral stem cells and hydrogels in periodontal regeneration

**Figure 4:**
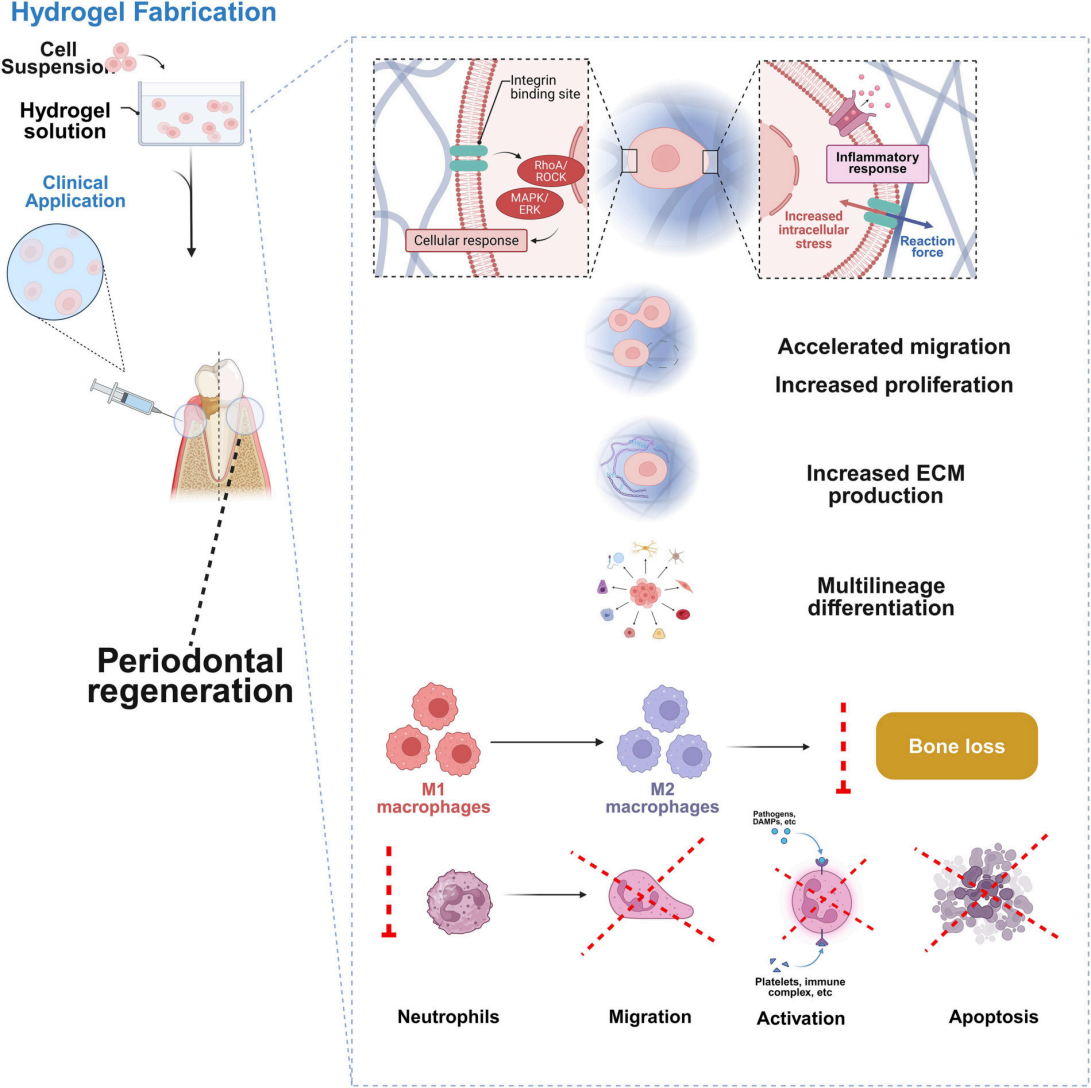
Implementation of oral stem cells-laden hydrogels in periodontal regeneration. This figure was created based on the tools provided by Biorender.com (accessed November 2, 2025).

The inclusion of oral MSCs into hydrogels creates an integrated foundation for tissue healing through the integration of gels' architectural and protective qualities with their ability to regenerate stem cells (Figure 4) (217, 218). This blend enhances cellular activity, optimizes immunological modulation, and ensures long-lasting biological efficacy, all of which are crucial for treating periodontitis (219). Researchers have discovered that oral MSCs in hydrogels significantly improve collagen accumulation and tissue epithelialization when compared to standard therapies, resulting in faster wound healing (220–222). Hydrogels are effective MSC carriers due to their unique features, including the capacity to transfer physiologically active substances. Hydrogels incorporating MSCs or their exosomes significantly enhance cellular recruitment and division, thereby accelerating re-epithelialization and wound healing. Additionally, the hydrogel-exosome complex activates key regeneration pathways, such as the PI3K/Akt pathway (197).

### 6.1 DFSCs laden hydrogels

DFSCs sheets exhibit high extracellular secretion capacity and efficiency in periodontal regeneration. DFSCs sheets produced from passage 4 cells outperformed DFSCs suspensions in terms of vitality and osteogenic transformation potential. Following 10 days of culturing, DFSCs sheets showed upregulation of osteogenic biomarkers. In comparison to the cell suspension, the cell sheet exhibited superior regenerative and paracrine capabilities *in vivo*. The modified DFSCs sheets revealed considerably greater concentrations of VEGF and angiopoietin-1 in comparison with DFSCs suspensions, as well as improved osteogenic initiation effects (223).

Tri-layered hydrogel formed of chitin-poly(lactic-co-glycolic acid) (PLGA) composite hydrogel. The first layer contained nanoinspired bioactive glass (nBG). The second layer contained fibroblast growth factor 2, and the third layer was loaded with nBG/platelet-rich plasma. It was biocompatible and promoted cementogenesis, fibrogenesis, and osteogenesis of human DFSCs both *in vitro* and *in vivo* (224). A 3D bioprinted biomimetic cell-laden bioink containing gelatin methacrylate/decellularized extracellular matrix (GelMA/dECM). The dECM exhibited strong immune-modulating properties while minimizing local inflammation *in vivo*. This hydrogel substantially promoted harmonious PDL fiber regeneration and enhanced bone mineralization *in vivo* (225).

### 6.2 DPSCs-laden hydrogels

Although deferoxamine (DFO) promotes angiogenesis during bone regeneration and wound healing, its effect on DPSCs driven angiogenesis remains uncertain. Here, DFO was loaded into gelatin-based microspheres (GMSs), and thermally responsive injectable hydrogels containing chitosan and collagen were created to allow for regulated DFO release. Due to its advantageous physical characteristics and biocompatibility, the DFO-GMS-laden hydrogel composite enabled prolonged DFO administration for up to 15 days. *In vitro*, DFO successfully induced tube formation, increased the release of molecules linked to angiogenesis, and encouraged DPSCs' movement (226).

In addition, Divband et al. (227), created new injectable hydrogels *in situ* using chitosan biguanidine and carboxymethylcellulose that were loaded with rhBMP-2 and VEGF. They investigated the impact of the fabricated hydrogels on the osteoblastic development of DPSCs. These hydrogels considerably boosted the growth of DPSCs and were non-toxic. Additionally, they displayed noticeably increased ALP, COL1α1, and OCN genetic and protein transcriptions (227).

Wang et al. (228), investigated hyaluronic acid (HA) and polyethylene glycol loaded with DPSCs' extracellular vesicles (EVs), which successfully relieved inflammation, expedited revascularization, and encouraged tissue mineralization *in vivo* (228).

GelMA-based biomaterials are frequently employed as scaffolds due to their biological compatibility, adjustable characteristics, and functional capacities. Nevertheless, the biological effects of its photo-crosslinking mechanism on MSCs, as well as stress-reduction methods, have yet to be fully investigated. Integrating DPSCs-CM into the GelMA hydrogel demonstrated increased cellular survival, growth, movement, and osteogenic transformation (229).

An injectable thermally responsive Cs/β-glycerophosphate/hydroxyapatite hydrogel can preserve typical adherent DPSCs cell shape, encourage fast multiplication, promote high cell viability, and enhance osteogenic activity (230).

### 6.3 GMSCs-laden hydrogels

It is found that alginate is a potential non-toxic platform for GMSCs encapsulation (231). Moreover, alginate hydrogel could promote osseointegration of GMSCs *in vitro* (231). Fawzy El-Sayed et al. (232) explored the regenerative capacity of GMSCs to reconstruct periodontal tissue when combined with HA-sECM that releases IL-1ra. GMSCs exhibited stem and MSCs properties. Interleukin (IL)-1ra-loaded and unloaded GMSCs/HA-sECM demonstrated increased clinical attachment level (CAL), decreased junctional epithelium (JE), and enhanced bleeding on probing (BOP) compared to negative controls (232). Wang et al. (76) developed a CS/ oxidized chondroitin sulfate (OCS) hydrogel by freeze-casting process to carry PDLSCs and GMSCs with the goal of inducing periodontal tissue regeneration. The PDLSCs and GMSCs loaded hydrogels demonstrated outstanding biocompatibility, more significant bone tissue healing, and generated more organized PDLs *in vivo* (76).

Furthermore, Ansari et al. (233) created an alginate/GelMA hydrogel formulation that encapsulates GMSCs. The GMSC-hydrogel could speed up wound healing by increasing collagen production, encouraging angiogenesis, and blocking local proinflammatory cytokines (233). Balaban et al. (120) verified that the local treatment of GMSCs in fibroin/chitosan oligosaccharide lactate hydrogel (F/COS) resulted in significant new bone growth and less lengthy junctional epithelium creation with well-organized PDLs and connective tissues (120).

Moshaverinia et al. (234) demonstrated that microencapsulation of PDLSCs and GMSCs in RGD-alginate hydrogel improved MSCs survival and osteogenic transformation *in vitro*. Moreover, PDLSCs loaded hydrogels could restore the damaged bone by stimulating the creation of mineralized tissue, while GMSCs had much inferior osteogenic differentiation competence (234). Pouraghaei Sevari et al. (235) developed alginate-whitlockite (WHMP) hydrogels, which showed enhanced elasticity without impacting the viability of GMSCs. Additionally, alginate-WHMP hydrogels stimulate the mitogen-activated protein kinase (MAPK) system, which regulates various osteogenic biomarkers in encapsulated GMSCs, particularly RUNX2 and osteocalcin (OCN). They could inhibit osteoclastic activity, likely thanks to the liberation of WHMP Mg^2+^ ions and GMSCs osteoprotegerin. GMSCs encapsulated in an osteogenic niche may improve bone repair *in vivo* (235). Kandalam et al. (167) employed a self-assembled hydrogel scaffold, PuraMatrix™ (PM), and/or BMP2 and loaded them with GMSCs. GMSCs-laden hydrogels could enhance bone regeneration upon *in vivo* application (167).

### 6.4 SCAPs-laden hydrogels

The good physical characteristics and biodegradability of a temperature-sensitive hydrogel have been demonstrated, making it an effective scaffold. Thermosensitive hydrogel in conjunction with lentiviral PDGF-BB loaded with SCAPS dramatically increased new bone growth and mineralization (236). Dutta et al. (237) demonstrated that 3D bioprinted temperature-sensitive poloxamer-407 (P407) hydrogels are non-toxic for encapsulating SCAPs, leading to high cellular survival and increased cell migratory potential. After 14 days of *in vitro* growth, the 3D hydrogels containing PAI-1 showed significantly higher levels of osteogenic biomarkers (237).

### 6.5 SHEDs laden-hydrogels

Chen et al. (238), created an injectable antimicrobial peptide-/(GelMA-AMP) hydrogel that contains hypoxia-inducible factor (HIF-1α), which promoted osteogenic transformation in SHEDs (238). Qu et al. develop SHEDs laden with metformin (MF)-loaded mesoporous silica nanospheres (MSNs)-/ (GelMA) photo-cured hydrogels. This bioactive hydrogel did not affect cell survival and showed significant activation of osteogenic-related genes (excluding OCN) (238).

### 6.6 PDSCs-laden hydrogels

Ivanov et al. (239), demonstrated that PDLSCs grown in a 3D collagen I hydrogel with dECM for 14 days exhibited phenotypic traits comparable to those of osteoblast-like cells. The discharge of bone-forming biomarkers (OC, OPN, and ALP) demonstrates this capacity. In 3D culture, the inclusion of fibronectin to the dECM promotes the most efficient conversion of PDLSCs into osteoblasts (240). PDLSC-laden GelMA and PEG dimethacrylate hydrogel can promote PDLSC survival and spreading (239). HydroMatrix™ (HydM) is a synthesized self-assembly peptide that can produce hydrogels with thermal or ion concentration variations. Nagy et al. (183) discovered that PDLSCs can attach, live, relocate, and multiply on HydM, and the gel promotes osteogenic transformation, encourages the viability of PDLSCs and their osteogenic transformation *in vitro*, as well as hastens osteogenesis *in vivo* (183).

### 6.7 BFPSCs

Saputra et al. (241) constructed a nanohydroxyapatite-chitosan (nHPA-CS) hydrogel injectable scaffold loaded with PRF and BFPSCs. The NHPA-CS scaffold develops a native microenvironment that stimulates BFPSCs' driven regenerative capacity, whereas PRF promotes osteogenic differentiation and multiplication of BFPSCs. The integration of nHPA-CS, platelet-rich fibrin (PRF), and BFPSCs exhibits osteoinductive capacity in patients with aggressive periodontitis (241). Bastami et al. (242) developed a 3D β-tricalcium phosphate (β-TCP)/gelatin/BMP2/chitosan (CS)/collagen composite and explored its impact on hBFPSCs. This hydrogel might serve not only as a structurally and physiologically suitable scaffolding, but also as an osteoinductive graft, supplying rhBMP2 during a therapeutic window for hBFPSC development into the osteoblast lineage (242).

## 7 Challenges of translating research from the laboratory to clinical practice

Oral stem cells loaded hydrogels encounter concerns such as cell viability and breakdown, inadequate mechanical characteristics, decomposition issues, and biocompatibility and antigenicity (243–245). These issues stem from the difficulty of tailoring the hydrogel's biomechanical features to the biological context of the stem cells while maintaining the cells' stability and functionality as the hydrogel breaks down and tissue grows (226, 228, 246).

The homogeneous distribution of molecules across the hydrogel network is crucial for preserving controlled characteristics and enhancing the functionality of the hydrogel (247). Effective dispersion methods and surface modifications are necessary to improve biocompatibility with the hydrogel matrix, thereby addressing this issue. Hydrogels must maintain their long-term stability to be used effectively (27, 248). The cytotoxicity and adaptability of hydrogels are crucial concerns, as some may have deleterious effects on stem cells or other cells (249, 250). Scalability and cost are essential factors to consider when evaluating the use of hydrogels in therapeutic applications (219). Large-scale production necessitates streamlining manufacturing processes while guaranteeing material quality and homogeneity (197). Cost-effective synthesis processes and raw material availability must be examined to ensure the feasibility and accessibility of these materials for broad use (251).

### 7.1 Scaling up

Limitations with employing stem cells include decreased survival of fewer cells, restricted division, and the stem cells' capability to differentiate. Although oral stem cells have demonstrated enhanced periodontal regeneration, there is still a lack of pre-clinical and clinical evidence, and more carefully thought-out studies with a bigger sample size are necessary to determine whether employing stem cells to boost periodontal healing is feasible (252). The effectiveness of stem cells in promoting periodontal regeneration is reduced when their sustainability is reduced as a result of late glycosylation byproducts in a hyperglycemic condition (253). DPSCs have shown the ability to mineralize. Indeed, it has been established that human DPSCs encourage osteogenesis both *in vitro* and *in vivo*. DPSCs' low ability to generate cementum raises doubts about their efficacy for periodontal regeneration. Despite these experiments showing oral stem cells' capacity for regeneration, the majority of them lack comprehensive quantitative methods to assess the cells' capacity for self-renewal, proliferation, and differentiation, particularly *in vivo* (87, 254, 255).

Furthermore, preceding their clinical use, the laboratory tests must address the subsequent concerns: 1) Significant apoptosis in cells at the transplantation site, thus the longevity and functionality of oral stem cells *in vivo* must be enhanced. 2) The relationship between implanted cells and the native cells must be investigated; 3) *In vivo* cell lineage mapping of implanted oral stem cells is essential to comprehend their destiny and activities. 4) Because some oral stem cells are frequently engaged in carcinoma, the biochemical pathways that enable oral stem cells to select between self-renewal, malignancy, and transformation should be thoroughly investigated (22, 256).

In addition, while these experiments are extremely helpful in discovering the characteristics of oral stem cells, they do not completely mirror the biological and pathological state of the injured tissues in the human body. Double-blind, randomized controlled studies are required to prove their genuine regeneration ability. Because clinical studies require a high quantity of clinical-grade cells within a short period, preservation and handling of oral stem cells are viable options for clinical use (256). After Hiroshima University established an institutional tooth bank in Japan in 2004, numerous biotechnology companies specializing in teeth banking have begun operations. With improved preservation technologies, regenerative dentistry could potentially act as a portal to an extensive spectrum of rejuvenating therapies by successfully tapping these beneficial stem cell reserves (257).

### 7.2 Regulatory hurdles

While the technical details of the banking procedure should worry an interested person, there is typically little information accessible from the public side of dental banks that enables people to distinguish between solutions. Their selection will most certainly be heavily affected by both regional accessibility and industry-specific, or more specifically, financial variations across banking centers (258). Additionally, prolonged stem cell preservation is not currently covered by medical insurance policies in the United States and is not a regular service in the majority of nations. A straight cost analysis may be useless because charges are liable to fluctuation, and there are many other types of plans available. However, as a rule of thumb, the initial processing may cost between $500 and $2,000 (US dollars), and the yearly maintenance can cost between $99 and $264. For a 20-year contract with no annual maintenance fees, several businesses offer a fixed fee of between $2,000 and $3,000 (259). In fact, this is Oothy's sole product, with the notion of preserving stem cells from deciduous teeth as a sustainable therapeutic investment. Therefore, choosing a service based solely on expense is undoubtedly a challenging and intimidating task, and it still fails to account for the specific services provided by each tooth bank. In this regard, NDPL, Oothy, and Stem-Save can collect a large number of teeth simultaneously. Still, BioEden and Tooth Bank do not charge for handling teeth until a satisfactory stem cell extraction is achieved. Some businesses provide full reimbursement if the entire procedure fails, while others offer credits for future teeth. Licensing and reliability are two additional unpredictable criteria. While every center guarantees security for cryopreservation operations, liquid nitrogen storage, and patient privacy, it is challenging for individual customers to compare centers based on these features. Eventually, it is realistic to expect dentists or other healthcare professionals to attract potential patients to the notion of oral stem cell banking and to suggest personalized banking services, which will have the most significant impact on any subsequent preference (260).

### 7.3 Ethical considerations

Despite the promising potentials of stem cells, numerous investigations are necessary to establish the safety and efficacy of these cells in periodontal applications. However, the standardized design and improvement of oral stem cell cryopreservation techniques must overcome significant hurdles, such as culture-driven variances, patient-associated variations, and the effects of culture medium additions. Only in this manner can we enhance and strengthen oral stem cells as potential therapeutic options for patients who have few or no other treatment options (261). These stem cells are becoming increasingly essential not just in dental care, where they play a crucial role in tissue regeneration and preservation, but also in other medical sectors, where they are gaining popularity. In summary, further examinations are required to confirm the seemingly regenerative ability of these stem cells. Still, it appears extremely promising to examine their regeneration capacities in a wide range of disorders, given that they are easily accessible, exist throughout life, and possess remarkable multipotency (262).

## 8 Future perspectives

Oral stem cells offer potential for numerous purposes, such as pharmaceutical testing and regenerative applications (233, 263). These applications need the production of exceptional stem cells in huge quantities (264).

The immunity-regulating properties of stem cells render them a promising treatment for periodontitis (265, 266). However, there is limited evidence of stem cell-based modulation of the immune system. Because periodontal defects are packed with calculus, bacterial biofilms, and plaque, it is exceedingly difficult to replicate highly contaminated environments in animal models (267). Additionally, oral stem cell-dependent immunomodulation pathways exhibit several differences. Several factors, including the origin of stem cells and the experimental methodology, can influence the quantity and quality of stem cells. The results of immunomodulation and regeneration might be affected by the subjects and methods used for stem cell extraction and transplantation (254, 266).

With structural difficulties, especially in furcation areas and small periodontal pockets, periodontal disease raises the probability of extraction of teeth and systemic health issues, necessitating sophisticated medication delivery methods. By infiltrating epithelial cells and evading intracellular inflammatory reactions, the bacterium *Porphyromonas gingivalis* accelerates the progression of the illness. Contemporary hydrogel delivery technologies do not sufficiently promote medication delivery and the removal of bacteria. Making use of chaotropic anions' ability to permeable membranes (268, 269).

Wang et al. (19) developed an iodide-functionalized gelatin/poly-L-lysine (PLL) hydrogel. This method displayed improved injectability, resulting in adequate pocket penetration and strong mucosal adherence. Significant amounts of iodide (5–10 mM) discharged at mucosal interfaces could enhance PLL trans-epithelial transport by membrane fluidization, boosting intracellular *P. gingivalis* clearance. Iodide levels (0.5–1.0 mM) inhibited inflammation while promoting osteogenesis *in vitro* and *in vivo*. This versatile device utilizes chaotropic ion interactions to substantially promote intracellular transport of antimicrobial peptides, enabling collaborative elimination of bacteria while simultaneously achieving immune modulation, osteogenesis, and MSCs survival, thereby contributing to targeted hydrogels for periodontal therapy (19).

Notwithstanding hurdles, as cell-laden hydrogel-based technology has evolved, substantial advancement has been achieved in stem cell multiplication and guided differentiation (233, 263). Many hydrogel features, such as biodegradable properties, durability, and porosity, perform critical roles in stem cell multiplication and differentiation (270, 271). Hydrogels' biochemical and physical features can be precisely tuned to mimic the natural environment in which diverse stem cells reside *in vivo* (272). As a result, various properties of hydrogels, including biodegradability, stiffness, shape, and metabolites, play important roles in determining their destiny and can impact stem cell differentiation and proliferation (170). These are important concerns to consider when dealing with hydrogels and stem cells (273). Hydrogels are an appealing option for regenerative applications; however, when conducting experiments with hydrogels, a variety of elements, characteristics, and metrics must be considered to achieve the desired outcomes (274). When employing hydrogels, the base material, whether natural or synthetic, should be chosen, and the overlaps and disparities should be evaluated. Additionally, the use of hybrid hydrogels and their accompanying properties must be investigated (249). The porosity of hydrogels should also be examined, and correlations between morphology and adhesion should be developed (221).

One should consider the likelihood of insufficient mechanical aspects and system consistency; while restricted, possible solutions to these issues may involve the introduction of peptides (275). A thorough examination of hydrogels would also include a study of the host's reaction to the treatment, particularly when employing hydrogels implanted with stem cells (276). Returning to the selection of hydrogels, the cellular response is a good indicator of which hydrogel to utilize. An ideal hydrogel is one that completely disintegrates while also allowing cells to grow in a manner that matches the native tissue perfectly, which is where the problem lies (277). As a result, tackling the issues related to cell-laden oral stem cells hydrogels necessitates a collaborative effort spanning various disciplines, including material science, biology, and engineering (278). By addressing these problems and highlighting future-oriented viewpoints, the integration of hydrogels and stem cells presents an opportunity to transform the field of regenerative healthcare (181, 279, 280). This can accelerate the advancement of novel treatment options for tissue repair and regeneration, leading to substantial advances in customized and personalized medicine (281–284).

## 9 Conclusion

Considering the increasing incidence of periodontitis and the potential risk it poses to retaining the entire dentition, the emphasis has shifted to microscopic PDL rebuilding to improve therapeutic outcomes for an increasing number of periodontal diseases. This paper focuses on oral stem cells, which have been recognized for their outstanding multipotency and immune-modulating properties. It provides a physiological summary as well as an explanation of how they are used in periodontal therapeutic applications. The primary mechanisms driving periodontal regeneration include restoring defective collagen metabolism, limiting tissue degradation, boosting regeneration of other damaged tissues, and promoting ordered PDL regeneration. Furthermore, this study gives a detailed overview of the application forms and recent developments in oral stem cells, highlighting their importance in the reconstruction of periodontal tissues in individuals with periodontitis. It describes potential investigation prospects in periodontal regeneration and offers a platform for exploratory investigations in PDL regeneration. Despite the significant improvement so far, numerous barriers still require consideration and improvement. A thorough comprehension of the processes regulating oral stem cells and their regeneration is still in its early stages of development. Moreover, the best option of oral stem cells for periodontal regeneration remains a topic of debate, prompting further exploration into diverse oral stem cell types, donor selection guidelines, and established techniques for cell collection and storage. In addition, disparities between experimental and clinical regenerative findings underscore the need for in-depth investigation of the basic processes, advancement of oral stem cell techniques, and the utilization of novel approaches such as bioactive factor combinations, innovative methods of administration, and specialized functional adjustments. Furthermore, it is crucial to investigate application strategies tailored to periodontal patients, enabling better alignment with therapeutic needs. To summarize, oral stem cells have tremendous promise for PDL regeneration because of their exceptional effectiveness, regeneration capability, and immune-modulating characteristics.

Periodontitis causes the alveolar bone to resorb and lose its connection gradually. Complete regeneration of periodontal tissue in both form and function is clinically challenging for individuals with severe periodontitis, making the condition particularly catastrophic for these patients. In the medical management of periodontitis, oral stem cell-loaded hydrogels can considerably accelerate periodontal reconstruction since they have the capacity to promote periodontal regeneration. Additionally, the hydrogels prevented the formation of periodontitis while hastening the recovery of healthy periodontium.

This review emphasizes the importance of functioning periodontium for patients with periodontitis, suggests additional investigation goals in periodontal regeneration, and offers an overview of fundamental periodontal regeneration investigations. It provides a concise yet informative summary, as well as a helpful reference for prospective studies in the associated fields.

## References

[1] Avetisyan A Markaryan M Rokaya D Tovani-Palone MR Zafar MS Khurshid Z Characteristics of periodontal tissues in prosthetic treatment with fixed dental prostheses. Molecules. (2021) 26(5):1331. 10.3390/molecules26051331 33801337 PMC7958327

[2] Isola G Polizzi A Serra S Boato M Sculean A. Relationship between periodontitis and systemic diseases: a bibliometric and visual study. Periodontology 2000. (2025):1–13. 10.1111/prd.12621 PMC12842847 39775963

[3] Zhang X Wang X Wu J Wang M Hu B Qu H The global burden of periodontal diseases in 204 countries and territories from 1990 to 2019. Oral Dis. (2024) 30(2):754–68. 10.1111/odi.14436 36367304

[4] Chen MX Zhong YJ Dong QQ Wong HM Wen YF. Global, regional, and national burden of severe periodontitis, 1990–2019: an analysis of the global burden of disease study 2019. J Clin Periodontol. (2021) 48(9):1165–88. 10.1111/jcpe.13506 34101223

[5] Tattar R da Costa BDC Neves VCM. The interrelationship between periodontal disease and systemic health: the interrelationship between periodontal disease and systemic health. Br Dent J. (2025) 239(2):103–8. 10.1038/s41415-025-8642-2 40715391 PMC12296551

[6] Corana M Baima G Iaderosa G Franco F Zhang J Berta GN Salivary proteomics for detecting novel biomarkers of periodontitis: a systematic review. J Periodontal Res. (2025) 60(7):633–55. 10.1111/jre.13357 39620241 PMC12371805

[7] Polizzi A Quinzi V Lo Giudice A Marzo G Leonardi R Isola G. Accuracy of artificial intelligence models in the prediction of periodontitis: a systematic review. JDR Clin Trans Res. (2024) 9(4):312–24. 10.1177/23800844241232318 38589339

[8] Karobari MI Siddharthan S Adil AH Khan MM Venugopal A Rokaya D Modifiable and non-modifiable risk factors affecting oral and periodontal health and quality of life in south Asia. Open Dent J. (2022) 16:e187421062209270. 10.2174/18742106-v16-e2209270

[9] Hu W Qian X Lin X Chen Q Wu Z Chen Z Zeolitic imidazolate frameworks exert bioenergetic modulation via the cAMP/PKA/CREB signaling pathway to accelerate periodontal regeneration. Chem Eng J. (2025) 526:171251. 10.1016/j.cej.2025.171251

[10] Fan Y Pei J Li X Qin Y Xu Y Ke M Construction of tissue-engineered vascular grafts with high patency by mimicking immune stealth and blocking TGF-β mediated endothelial-to-mesenchymal transition. Compos B Eng. (2023) 251:110487. 10.1016/j.compositesb.2022.110487

[11] Zhou Z-Y Li Z-B Shi N-S Feng S-S Han Y-R Liu M-T Metal-polyphenol network-engineered mesenchymal stem cell-derived exosome mimetics mediate inflammatory/immune regulation for enhanced periodontal tissue regeneration. Biomaterials. (2025) 327:123696. 10.1016/j.biomaterials.2025.123696 41005081

[12] Ma Y Xie L Yang B Tian W. Three-dimensional printing biotechnology for the regeneration of the tooth and tooth-supporting tissues. Biotechnol Bioeng. (2019) 116(2):452–68. 10.1002/bit.26882 30475386

[13] Zheng X Xie T Sun S Sun L. Application of periodontal tissue regeneration combined with orthodontics in oral prosthodontics and its influence and significance on the expressions of IL-1β, TNF-α and IL-5 in periodontal tissue. Biotechnol Genet Eng Rev. (2024) 40(3):2295–307. 10.1080/02648725.2023.2199242 37036953

[14] Guo Q Ji X Zhang L Liu X Wang Y Liu Z Differences in the response of normal oral mucosa, oral leukoplakia, oral squamous cell carcinoma-derived mesenchymal stem cells, and epithelial cells to photodynamic therapy. J Photochem Photobiol, B. (2024) 255:112907. 10.1016/j.jphotobiol.2024.112907 38677259

[15] Di T Feng C Wang L Xu J Du Y Cheng B Enhancing vasculogenesis in dental pulp development: DPSCs-ECs communication via FN1-ITGA5 signaling. Stem Cell Rev Rep. (2024) 20(4):1060–77. 10.1007/s12015-024-10695-6 38418738 PMC11087358

[16] Long L Zhang C He Z Liu O Yang H Fan Z. LncRNA NR_045147 modulates osteogenic differentiation and migration in PDLSCs via ITGB3BP degradation and mitochondrial dysfunction. Stem Cells Transl Med. (2025) 14(2):szae088. 10.1093/stcltm/szae088 39674578 PMC11878762

[17] Yadav P Vats R Wadhwa S Bano A Namdev R Gupta M Enhancing proliferation of stem cells from human exfoliated deciduous teeth (SHED) through hTERT expression while preserving stemness and multipotency. Stem Cell Rev Rep. (2024) 20(7):1902–14. 10.1007/s12015-024-10746-y 38878252

[18] Kobayashi Y Nouet J Baljinnyam E Siddiqui Z Fine DH Fraidenraich D iPSC-derived cranial neural crest-like cells can replicate dental pulp tissue with the aid of angiogenic hydrogel. Bioact Mater. (2022) 14:290–301. 10.1016/j.bioactmat.2021.11.014 35310357 PMC8897656

[19] Wang R Zhou J Li X Xiong D Huang D Shi Y Chaotropic Ion-tuned protein/peptide hydrogel with enhanced trans-barrier delivery for effective periodontitis management via pathogen clearance, immunomodulation, and MSC preservation. Biomaterials. (2025) 327:123781. 10.1016/j.biomaterials.2025.123781 41115392

[20] Swetha G Priyangha PT Chithra S. Formulation of a novel polymeric hydrogel membrane for periodontal tissue regeneration using tricalcium phosphate-alginate reinforcement. Cureus. (2024) 16(4):e57844. 10.7759/cureus.57844 38721191 PMC11078324

[21] Luo Q Yang Y Ho C Li Z Chiu W Li A Dynamic hydrogel–metal–organic framework system promotes bone regeneration in periodontitis through controlled drug delivery. J Nanobiotechnol. (2024) 22(1):287. 10.1186/s12951-024-02555-9 PMC11129436 38797862

[22] Liu L Wen Y Chen L Li M Yu J Tian W Xenogenous implanted dental follicle stem cells promote periodontal regeneration through inducing the N2 phenotype of neutrophils. Stem Cell Res Ther. (2024) 15(1):270. 10.1186/s13287-024-03882-2 39183362 PMC11346187

[23] Zhang S Zhou H Liu X Wu Y Yang S Yin J Ceo_2_ nanozyme-engineered dental pulp mesenchymal stem cells for periodontal regeneration. ACS Appl Nano Mater. (2025) 8(18):9295–311. 10.1021/acsanm.5c00622

[24] Liang L Wang L Liao Z Ma L Wang P Zhao J High-yield nanovesicles extruded from dental follicle stem cells promote the regeneration of periodontal tissues as an alternative of exosomes. J Clin Periodontol. (2024) 51(10):1395–407. 10.1111/jcpe.14036 38951121

[25] Yang Y Xu C Xu S Li Y Chen KE Yang T Injectable hydrogels activated with copper sulfide nanoparticles for enhancing spatiotemporal sterilization and osteogenesis in periodontal therapy. Biomater Sci. (2025) 13(6):1434–48. 10.1039/D3BM02134C 38711336

[26] Suciu T-S Feștilă D Berindan-Neagoe I Nutu A Armencea G Aghiorghiesei AI Circular RNA-mediated regulation of oral tissue-derived stem cell differentiation: implications for oral medicine and orthodontic applications. Stem Cell Rev Rep. (2024) 20(3):656–71. 10.1007/s12015-024-10683-w 38279054 PMC10984898

[27] Ming L Qu Y Wang Z Dong L Li Y Liu F Small extracellular vesicles laden oxygen-releasing thermosensitive hydrogel for enhanced antibacterial therapy against anaerobe-induced periodontitis alveolar bone defect. ACS Biomater Sci Eng. (2024) 10(2):932–45. 10.1021/acsbiomaterials.3c00493 38275448

[28] Salazar NJP Soe ZC Nan DN Vongsutilers V Trachoo V Osathanon T Enhanced neurogenic differentiation of human dental pulp stem cells via BDNF-loaded oxidized alginate hydrogel. J Dent. (2025) 163:106185. 10.1016/j.jdent.2025.106185 41109553

[29] Alipour M Ghorbani M Johari khatoonabad M Aghazadeh M. A novel injectable hydrogel containing polyetheretherketone for bone regeneration in the craniofacial region. Sci Rep. (2023) 13(1):864. 10.1038/s41598-022-23708-6 36650203 PMC9845302

[30] Wang W Zhu Y Li J Geng T Jia J Wang X Bioprinting EphrinB2-modified dental pulp stem cells with enhanced osteogenic capacity for alveolar bone engineering. Tissue Eng, Part A. (2023) 29(7-8):244–55. 10.1089/ten.tea.2022.0180 36606680

[31] Ying Y Huang Z Tu Y Wu Q Li Z Zhang Y A shear-thinning, ROS-scavenging hydrogel combined with dental pulp stem cells promotes spinal cord repair by inhibiting ferroptosis. Bioact Mater. (2023) 22:274–90. 10.1016/j.bioactmat.2022.09.019 36263097 PMC9556860

[32] Jiang Y Chen Y Ge L Wang L Wang L Pathak JL. Multi-prospects of bacterial extracellular vesicles in immune modulation, inflammation regulation, and periodontitis treatment. Nano Today. (2024) 55:102210. 10.1016/j.nantod.2024.102210

[33] Zhang S Jiao Y Liu S Liu Y Liu Y. Effects of Porphyromonas gingivalis outer membrane vesicles (OMVs) on macrophages in periodontitis. Oral Dis. (2025) 31(7):1973–81. 10.1111/odi.15264 39887837

[34] Corbella S Calciolari E Alberti A Donos N Francetti L. Systematic review and meta-analysis on the adjunctive use of host immune modulators in non-surgical periodontal treatment in healthy and systemically compromised patients. Sci Rep. (2021) 11(1):12125. 10.1038/s41598-021-91506-7 34108528 PMC8190303

[35] Meenakshi SS Sankari M. Effectiveness of chitosan nanohydrogel as a bone regenerative material in intrabony defects in patients with chronic periodontitis: a randomized clinical trial. J Adv Oral Res. (2021) 12(2):222–8. 10.1177/2320206821998574

[36] Xiang J Zhou X Xia Z Zhang Z Xu K Hu S All-trans retinoic acid-functionalized injectable hydrogel with immunomodulation, osteogenesis and antibacterial capability for periodontitis therapy. Chem Eng J. (2024) 500:156915. 10.1016/j.cej.2024.156915

[37] Zeng X Wang X Guan X Feng X Lu R Meng H. The long-term effect of periodontitis treatment on changes in blood inflammatory markers in patients with generalized aggressive periodontitis. J Periodontal Res. (2024) 59(4):689–97. 10.1111/jre.13251 38501229

[38] Ruan D Wu C Zhang Y Zhang Y. LncRNA LOXL1-AS1 inhibits proliferation of PDLSCs and downregulates IL-1β in periodontitis patients. J Periodontal Res. (2022) 57(2):324–31. 10.1111/jre.12962 34910833

[39] Xia S Jing R Shi M Yang Y Feng M Deng L BBR Affects macrophage polarization via inhibition of NF-κB pathway to protect against T2DM-associated periodontitis. J Periodontal Res. (2024) 59(4):728–37. 10.1111/jre.13246 38501225

[40] Kosho MXF Ciurli A Giera M Neefjes J Loos BG. Metabolomic profiles of oral rinse samples to distinguish severe periodontitis patients from non-periodontitis controls. J Periodontal Res. (2025) 60(8):762–74. 10.1111/jre.13379 40083241 PMC12476085

[41] Saliem SS Bede SY Abdulkareem AA Abdullah BH Milward MR Cooper PR. Gingival tissue samples from periodontitis patients demonstrate epithelial–mesenchymal transition phenotype. J Periodontal Res. (2023) 58(2):247–55. 10.1111/jre.13086 36575609

[42] Yang X Tao X Qi W Liu Z Wang Y Han Q TLR-4 targeting contributes to the recovery of osteoimmunology in periodontitis. J Periodontal Res. (2021) 56(4):782–8. 10.1111/jre.12877 33729573

[43] Jiang Q Zhao Y Shui Y Zhou X Cheng L Ren B Interactions between neutrophils and periodontal pathogens in late-onset periodontitis. Front Cell Infect Microbiol. (2021) 11:627328. 10.3389/fcimb.2021.627328 33777839 PMC7994856

[44] Vitkov L Muñoz LE Schoen J Knopf J Schauer C Minnich B Neutrophils orchestrate the periodontal pocket. Front Immunol. (2021) 12:788766. 10.3389/fimmu.2021.788766 34899756 PMC8654349

[45] Sansores-España LD Melgar-Rodríguez S Vernal R Carrillo-Ávila BA Martínez-Aguilar VM Díaz-Zúñiga J. Neutrophil N1 and N2 subsets and their possible association with periodontitis: a scoping review. Int J Mol Sci. (2022) 23(20):12068. 10.3390/ijms232012068 36292925 PMC9603394

[46] Zhang J Ding Q Wang AX Lin M Yu N Moss K Type I interferon protects against bone loss in periodontitis by mitigating an interleukin (IL)-17-neutrophil axis. Life Sci. (2025) 371:123559. 10.1016/j.lfs.2025.123559 40086745 PMC12094266

[47] Medara N Lenzo JC Walsh KA O’Brien-Simpson NM Reynolds EC Darby IB. Peripheral T helper cell profiles during management of periodontitis. J Clin Periodontol. (2021) 48(1):77–91. 10.1111/jcpe.13389 33051896

[48] Ghasemi S Mortezagholi B Movahed E Ghaedi A Bazrgar A Abdolalizadehil S Systematic review and meta-analysis of the association of neutrophil-to-lymphocyte ratio and platelet-to-lymphocyte ratio with periodontitis. Eur J Med Res. (2024) 29(1):581. 10.1186/s40001-024-02175-x 39696713 PMC11657933

[49] Kou Y Jiang Y Liu S Yang P Lu Y Liu H Regulatory T cells showed characteristics of T helper-17 (Th17) cells in mice periodontitis model. Oral Dis. (2023) 29(3):1149–62. 10.1111/odi.14072 34741371

[50] Li Y Chen Y Cai G Ni Q Geng Y Wang T Roles of trained immunity in the pathogenesis of periodontitis. J Periodontal Res. (2023) 58(5):864–73. 10.1111/jre.13158 37424315

[51] Jiang Y Song B Brandt BW Cheng L Zhou X Exterkate RAM Comparison of red-complex bacteria between saliva and subgingival plaque of periodontitis patients: a systematic review and meta-analysis. Front Cell Infect Microbiol. (2021) 11:727732. 10.3389/fcimb.2021.727732 34692561 PMC8531218

[52] Kim HY Song MK Gho YS Kim HH Choi BK. Extracellular vesicles derived from the periodontal pathogen Filifactor alocis induce systemic bone loss through toll-like receptor 2. J Extracell Vesicles. (2021) 10(12):e12157. 10.1002/jev2.12157 34648247 PMC8516034

[53] Tang M Wang G Li J Wang Y Peng C Chang X Flavonoid extract from propolis alleviates periodontitis by boosting periodontium regeneration and inflammation resolution via regulating TLR4/MyD88/NF-κB and RANK/NF-κB pathway. J Ethnopharmacol. (2024) 319:117324. 10.1016/j.jep.2023.117324 37852336

[54] Muhssin SA Akram HM. Assessment of salivary levels of the RANKL and RANK in patients with healthy gingiva on reduced periodontium versus periodontitis: an analytical cross-sectional study. Dent Hypotheses. (2023) 14(2):49–51. 10.4103/denthyp.denthyp_17_23

[55] Deng J Lu C Zhao Q Chen K Ma S Li Z. The Th17/treg cell balance: crosstalk among the immune system, bone and microbes in periodontitis. J Periodontal Res. (2022) 57(2):246–55. 10.1111/jre.12958 34878170

[56] Yang D He D Yang F Meng X Zheng K Lin H Advances in harnessing biological macromolecules for periodontal tissue regeneration: a review. Int J Biol Macromol. (2025) 311:144031. 10.1016/j.ijbiomac.2025.144031 40345296

[57] Taymour N Haque MA Atia GAN Mohamed SZ Rokaya D Bajunaid SM Nanodiamond: a promising carbon-based nanomaterial for therapeutic and regenerative dental applications. ChemistrySelect. (2024) 9(35):e202401328. 10.1002/slct.202401328

[58] Atia GA Rashed F Taher ES Cho S-G Dayem AA Soliman MM Challenges of therapeutic applications and regenerative capacities of urine based stem cells in oral, and maxillofacial reconstruction. Biomed Pharmacother. (2024) 177:117005. 10.1016/j.biopha.2024.117005 38945084

[59] Chen Y Gao F Liu Q Yuan S Yu H Guo Y Preparation and formation mechanism study of antibiofilm coating based on phase transition of glutenin. Biomacromolecules. (2024) 25(8):5008–18. 10.1021/acs.biomac.4c00422 38956952

[60] Liu S Wang W Wu P Chen Z Pu W Li L Pathogenesis-guided engineering of multi-bioactive hydrogel co-delivering inflammation-resolving nanotherapy and pro-osteogenic protein for bone regeneration. Adv Funct Mater. (2023) 33(32):2301523. 10.1002/adfm.202301523

[61] Atia GAN Mohamed SZ Halim HA Ghobashy MM Foda T Shalaby HK Advances in bioceramic silicates for therapeutic, and regenerative dentofacial reconstruction. Ceram Int. (2024) 50(13):22184–208. 10.1016/j.ceramint.2024.04.035

[62] Abdel Nasser Atia G Shalaby HK Zehravi M Ghobashy MM Ahmad Z Khan FS Locally applied repositioned hormones for oral bone and periodontal tissue engineering: a narrative review. Polymers (Basel). (2022) 14(14):2964. 10.3390/polym14142964 35890740 PMC9319147

[63] Taymour N Ali MAM Taher ES Atia GA Abdeen A Chaudhary AA Functionalized nanodiamonds in dentistry: multifunctional frontiers for oral and maxillofacial regeneration. J Drug Deliv Sci Technol. (2025) 114:107448. 10.1016/j.jddst.2025.107448

[64] Atia GA Abdal Dayem A Taher ES Alghonemy WY Cho S-G Aldarmahi AA Urine-derived stem cells: a sustainable resource for advancing personalized medicine and dental regeneration. Front Bioeng Biotechnol. (2025) 13:1571066. 10.3389/fbioe.2025.1571066 40357329 PMC12066649

[65] Alkandari M Barai P Atia GAN Mohamed SZ Ghobashy MM Shalaby HK Bioactive functionalized chitosan thermo-responsive hydrogels as promising platforms for therapeutic, regenerative oral, and maxillofacial applications. Biotechnol J. (2025) 20(1):e202400653. 10.1002/biot.202400653 39865415

[66] El-Nablaway M Rashed F Taher ES Abdeen A Taymour N Soliman MM Prospective and challenges of locally applied repurposed pharmaceuticals for periodontal tissue regeneration. Front Bioeng Biotechnol. (2024) 12:1400472. 10.3389/fbioe.2024.1400472 39605747 PMC11600316

[67] Ren J Fok MR Zhang Y Han B Lin Y. The role of non-steroidal anti-inflammatory drugs as adjuncts to periodontal treatment and in periodontal regeneration. J Transl Med. (2023) 21(1):149. 10.1186/s12967-023-03990-2 36829232 PMC9960225

[68] Gao P Kajiya M Motoike S Ikeya M Yang J. Application of mesenchymal stem/stromal cells in periodontal regeneration: opportunities and challenges. Jpn Dent Sci Rev. (2024) 60:95–108. 10.1016/j.jdsr.2024.01.001 38314143 PMC10837070

[69] Liu S Wang W Chen Z Wu P Pu W Li G An osteoimmunomodulatory biopatch potentiates stem cell therapies for bone regeneration by simultaneously regulating IL-17/ferroptosis signaling pathways. Adv Sci. (2024) 11(35):2401882. 10.1002/advs.202401882 PMC11425236 39024121

[70] Kaasalainen M Zhang R Vashisth P Birjandi AA S’Ari M Martella DA Lithiated porous silicon nanowires stimulate periodontal regeneration. Nat Commun. (2024) 15(1):487. 10.1038/s41467-023-44581-5 38216556 PMC10786831

[71] Polizzi A Leanza Y Belmonte A Grippaudo C Leonardi R Isola G. Impact of hyaluronic acid and other Re-epithelializing agents in periodontal regeneration: a molecular perspective. Int J Mol Sci. (2024) 25(22):12347. 10.3390/ijms252212347 39596411 PMC11594871

[72] Shirbhate U Bajaj P. Third-generation platelet concentrates in periodontal regeneration: gaining ground in the field of regeneration. Cureus. (2022) 14(8):e28072. 10.7759/cureus.28072 36127983 PMC9477433

[73] Khalil MA Sonbol FI Al-Madboly LA Aboshady TA Alqurashi AS Ali SS. Exploring the therapeutic potentials of exopolysaccharides derived from lactic acid bacteria and bifidobacteria: antioxidant, antitumor, and periodontal regeneration. Front Microbiol. (2022) 13:803688. 10.3389/fmicb.2022.803688 35547125 PMC9082500

[74] Xu X Chen Z Xiao L Xu Y Xiao N Jin W Nanosilicate-functionalized nanofibrous membrane facilitated periodontal regeneration potential by harnessing periodontal ligament cell-mediated osteogenesis and immunomodulation. J Nanobiotechnol. (2023) 21(1):223. 10.1186/s12951-023-01982-4 PMC10339597 37443072

[75] De Ry SP Roccuzzo A Lang NP Sculean A Salvi GE. Long-term clinical outcomes of periodontal regeneration with enamel matrix derivative: a retrospective cohort study with a mean follow-up of 10 years. J Periodontol. (2022) 93(4):548–59. 10.1002/JPER.21-0347 34258767 PMC9373923

[76] Wang W Wang A Hu G Bian M Chen L Zhao Q Potential of an aligned porous hydrogel scaffold combined with periodontal ligament stem cells or gingival mesenchymal stem cells to promote tissue regeneration in rat periodontal defects. ACS Biomater Sci Eng. (2023) 9(4):1961–75. 10.1021/acsbiomaterials.2c01440 36942823

[77] de Souza Araújo IJ Perkins RS Ibrahim MM Huang GTJ Zhang W. Bioprinting PDLSC-laden collagen scaffolds for periodontal ligament regeneration. ACS Appl Mater Interfaces. (2024) 16(44):59979–90. 10.1021/acsami.4c13830 39467547 PMC11551894

[78] Liang X Gong Y Bai L Ahmadi S Yu M Zhang Z Assessing bone regeneration potential of 3D scaffold-free cell pellets from periodontal ligament and bone marrow stem cells. BMC Biotechnol. (2025) 25(1):55. 10.1186/s12896-025-00983-5 40597198 PMC12210542

[79] Liang X Zhang Z Fang S Elayah SA Bai L Ahmadi S Three-dimensional scaffold-free periodontal ligament stem cell pellets for alveolar ridge preservation: an *in vitro* and *in vivo* study. BMC Oral Health. (2025) 25(1):1227. 10.1186/s12903-025-06495-0 40696336 PMC12281866

[80] Tan KL Chia WC How CW Tor YS Show PL Looi QHD Benchtop isolation and characterisation of small extracellular vesicles from human mesenchymal stem cells. Mol Biotechnol. (2021) 63(9):780–91. 10.1007/s12033-021-00339-2 34061307

[81] Li Y Duan X Chen Y Liu B Chen G. Dental stem cell-derived extracellular vesicles as promising therapeutic agents in the treatment of diseases. Int J Oral Sci. (2022) 14(1):2. 10.1038/s41368-021-00152-2 34980877 PMC8724288

[82] Wen W Pang Y Tian Y Xu C Wang J Wu Y Osteogenic mesenchymal stem cells/progenitors in the periodontium. Oral Dis. (2024) 30(3):914–20. 10.1111/odi.14507 36648363

[83] Tóth F Tőzsér J Hegedűs C. Effect of inducible BMP-7 expression on the osteogenic differentiation of human dental pulp stem cells. Int J Mol Sci. (2021) 22(12):6182. 10.3390/ijms22126182 34201124 PMC8229115

[84] Wei X-L Luo L Chen M-Z Zhou J Lan B-Y Ma X-M Temporospatial expression of neuropeptide substance P in dental pulp stem cells during odontoblastic differentiation *in vitro* and reparative dentinogenesis *in vivo*. J Endod. (2023) 49(3):276–85. 10.1016/j.joen.2022.12.006 36549466

[85] Ma L Huang Z Wu D Kou X Mao X Shi S. CD146 Controls the quality of clinical grade mesenchymal stem cells from human dental pulp. Stem Cell Res Ther. (2021) 12(1):488. 10.1186/s13287-021-02559-4 34461987 PMC8404346

[86] Pan G Zhou Q Pan C Zhang Y. The impact of the VEGF/VEGFR2/PI3K/AKT signaling axis on the proliferation and migration abilities of human dental pulp stem cells. Cell Biochem Biophys. (2024) 82(3):2787–95. 10.1007/s12013-024-01394-7 38987441

[87] Xia J Zhang Z Weng J Zhu G Xu Z Zeng L Apoptotic extracellular vesicles derived from human dental pulp stem cells facilitate periodontal tissue regeneration. BMC Oral Health. (2025) 25(1):1150. 10.1186/s12903-025-06531-z 40646544 PMC12255120

[88] Chauca-Bajaña L Velasquez-Ron B Tomás-Carmona I Camacho-Alonso F Pérez-Jardón A Pérez-Sayáns M. Regeneration of periodontal bone defects with mesenchymal stem cells in animal models. Systematic review and meta-analysis. Odontology. (2023) 111(1):105–22. 10.1007/s10266-022-00725-5 35788845 PMC9810679

[89] Fageeh HN. Preliminary evaluation of proliferation, wound healing properties, osteogenic and chondrogenic potential of dental pulp stem cells obtained from healthy and periodontitis affected teeth. Cells. (2021) 10(8):2118. 10.3390/cells10082118 34440887 PMC8393753

[90] Bringel M Zalaf BR de Oliveira B Oliveira BLS Silveira ABV de Camargo MR Characterization and analysis of stem cells from human exfoliated deciduous teeth of children with and without cleft lip and palate. J Cleft Lip Palate Craniofacial Anomalies. (2025) 12(2):88–97. 10.4103/jclpca.jclpca_12_25

[91] Sugiaman VK Djuanda R Pranata N Naliani S Demolsky WL Jeffrey J. Tissue engineering with stem cell from human exfoliated deciduous teeth (SHED) and collagen matrix, regulated by growth factor in regenerating the dental pulp. Polymers (Basel). (2022) 14(18):3712. 10.3390/polym14183712 36145860 PMC9503223

[92] de Oliveira Lisboa M Selenko AH Hochuli AHD Senegaglia AC Fracaro L Brofman PRS. The influence of fetal bovine serum concentration on stemness and neuronal differentiation markers in stem cells from human exfoliated deciduous teeth. Tissue Cell. (2024) 91:102571. 10.1016/j.tice.2024.102571 39353229

[93] Vu HT Han M-R Lee J-H Kim J-S Shin J-S Yoon J-Y Investigating the effects of conditioned media from stem cells of human exfoliated deciduous teeth on dental pulp stem cells. Biomedicines. (2022) 10(4):906. 10.3390/biomedicines10040906 35453661 PMC9027398

[94] Sordi MB Curtarelli RB da Silva IT Fongaro G Benfatti CAM de Souza Magini R Effect of dexamethasone as osteogenic supplementation in *in vitro* osteogenic differentiation of stem cells from human exfoliated deciduous teeth. J Mater Sci: Mater Med. (2021) 32(1):1. 10.1007/s10856-020-06475-6 33469820 PMC7815568

[95] Kato M Tsunekawa S Nakamura N Miura-Yura E Yamada Y Hayashi Y Secreted factors from stem cells of human exfoliated deciduous teeth directly activate endothelial cells to promote all processes of angiogenesis. Cells. (2020) 9(11):2385. 10.3390/cells9112385 33142678 PMC7693657

[96] Zhou F Guo J Dong Z Zhao W He X Wu M SHED aggregate derived exosomes− shuttled miR-222 promotes the angiogenic properties of periodontal ligament stem cells and enhances periodontal regeneration. Trans Dental Res. (2025) 1(3):100032. 10.1016/j.tdr.2025.100032

[97] Gao X Shen Z Guan M Huang Q Chen L Qin W Immunomodulatory role of stem cells from human exfoliated deciduous teeth on periodontal regeneration. Tissue Eng, Part A. (2018) 24(17-18):1341–53. 10.1089/ten.tea.2018.0016 29652608

[98] Pinho LC Santos C Fernandes MH Colaço B. Canine periodontal ligament stem cells as a tool for periodontal regeneration. Res Vet Sci. (2025) 193:105787. 10.1016/j.rvsc.2025.105787 40614412

[99] Wada N Tomokiyo A Gronthos S Bartold PM. Immunomodulatory properties of PDLSC and relevance to periodontal regeneration. Curr Oral Health Rep. (2015) 2(4):245–51. 10.1007/s40496-015-0062-y

[100] Zhang D Lin W Jiang S Deng P Liu L Wang Q Lepr-expressing PDLSCs contribute to periodontal homeostasis and respond to mechanical force by Piezo1. Adv Sci. (2023) 10(29):2303291. 10.1002/advs.202303291 PMC10582421 37553778

[101] Xu F Zhang Z Fang S Bai L Gong Y Liang X Autocrine TGF-β1 in periodontal ligament-derived stem cell pellets enhances periodontal regeneration in class II furcation defects of canine models. Adv Healthcare Mater. (2025):e02553. 10.1002/adhm.202502553 40981603

[102] Liu Z Li Q Wang X Wu Y Zhang Z Mao J Proanthocyanidin enhances the endogenous regeneration of alveolar bone by elevating the autophagy of PDLSCs. J Periodontal Res. (2023) 58(6):1300–14. 10.1111/jre.13186 37715945

[103] Qu G Li Y Chen L Chen Q Zou D Yang C Comparison of osteogenic differentiation potential of human dental-derived stem cells isolated from dental pulp, periodontal ligament, dental follicle, and alveolar bone. Stem Cells Int. (2021) 2021(1):6631905. 10.1155/2021/6631905 33927769 PMC8049831

[104] Xu J Lin Y Tian M Li X Yin Y Li Q Periodontal ligament stem cell-derived extracellular vesicles enhance tension-induced osteogenesis. ACS Biomater Sci Eng. (2022) 9(1):388–98. 10.1021/acsbiomaterials.2c00717 36538768

[105] You J Zhang Q Qian L Shi Z Wang X Jia L Antibacterial periodontal ligament stem cells enhance periodontal regeneration and regulate the oral microbiome. Stem Cell Res Ther. (2024) 15(1):334. 10.1186/s13287-024-03939-2 39334342 PMC11437971

[106] Zhu Y Ge X Chen Z Chen T Wu Y Wen H METTL3-m6A Methylase regulates the osteo-/odontogenic potential of stem cells from apical papilla via NFIC in apical periodontitis. Exp Cell Res. (2025) 448:114576. 10.1016/j.yexcr.2025.114576 40280320

[107] Santos A Spigariol KS Santos LM Holzhausen M Sipert CR. Immunomodulatory effects of apical papilla cells on periodontal ligament fibroblasts stimulated with Escherichia coli lipopolysaccharide: an *in vitro* study. J Appl Oral Sci. (2025) 33:e20240338. 10.1590/1678-7757-2024-0338 40105577 PMC11869941

[108] Li G Han N Zhang X Yang H Cao Y Wang S Local injection of allogeneic stem cells from apical papilla enhanced periodontal tissue regeneration in minipig model of periodontitis. BioMed Res Int. (2018) 2018(1):3960798. 10.1155/2018/3960798 30112386 PMC6077668

[109] Chrepa V Pitcher B Henry MA Diogenes A. Survival of the apical papilla and its resident stem cells in a case of advanced pulpal necrosis and apical periodontitis. J Endod. (2017) 43(4):561–7. 10.1016/j.joen.2016.09.024 28190588

[110] Liu J Wang L Liu W Li Q Jin Z Jin Y. Dental follicle cells rescue the regenerative capacity of periodontal ligament stem cells in an inflammatory microenvironment. PLoS One. (2014) 9(10):e108752. 10.1371/journal.pone.0108752 25275580 PMC4183515

[111] Gao Y He X Deng Y Wang X Shen J Xu W Human dental follicle cell-derived conditioned media enhance periodontal regeneration by regulating the osteogenic differentiation and inflammation of periodontal ligament stem cells and macrophage polarization. Mol Cell Biochem. (2025) 480(7):4431–48. 10.1007/s11010-025-05260-1-18 40175780

[112] Kotova AV Lobov AA Dombrovskaya JA Sannikova VY Ryumina NA Klausen P Comparative analysis of dental pulp and periodontal stem cells: differences in morphology, functionality, osteogenic differentiation and proteome. Biomedicines. (2021) 9(11):1606. 10.3390/biomedicines9111606 34829835 PMC8616025

[113] Morsczeck C Götz W Schierholz J Zeilhofer F Kühn U Möhl C Isolation of precursor cells (PCs) from human dental follicle of wisdom teeth. Matrix Biol. (2005) 24(2):155–65. 10.1016/j.matbio.2004.12.004 15890265

[114] Han C Yang Z Zhou W Jin F Song Y Wang Y Periapical follicle stem cell: a promising candidate for cementum/periodontal ligament regeneration and bio-root engineering. Stem Cells Dev. (2010) 19(9):1405–15. 10.1089/scd.2009.0277 19995154

[115] Yildirim S Zibandeh N Genc D Ozcan EM Goker K Akkoc T. The comparison of the immunologic properties of stem cells isolated from human exfoliated deciduous teeth, dental pulp, and dental follicles. Stem Cells Int. (2016) 2016(1):4682875. 10.1155/2016/4682875 26770205 PMC4684887

[116] Dave JR Chandekar SS Behera S Desai KU Salve PM Sapkal NB Human gingival mesenchymal stem cells retain their growth and immunomodulatory characteristics independent of donor age. Sci Adv. (2022) 8(25):eabm6504. 10.1126/sciadv.abm6504 35749495 PMC9232118

[117] Hu Y Wang Z Fan C Gao P Wang W Xie Y Human gingival mesenchymal stem cell-derived exosomes cross-regulate the Wnt/β-catenin and NF-κB signalling pathways in the periodontal inflammation microenvironment. J Clin Periodontol. (2023) 50(6):796–806. 10.1111/jcpe.13798 36843393

[118] Sun J Wang Z Liu P Hu Y Li T Yang J Exosomes derived from human gingival mesenchymal stem cells attenuate the inflammatory response in periodontal ligament stem cells. Front Chem. (2022) 10:863364. 10.3389/fchem.2022.863364 35464198 PMC9019468

[119] Mitrano TI Grob MS Carrion F Nova-Lamperti E Luz PA Fierro FS Culture and characterization of mesenchymal stem cells from human gingival tissue. J Periodontol. (2010) 81(6):917–25. 10.1902/jop.2010.090566 20450355

[120] Balaban YE Akbaba S Bozkurt SB Buyuksungur A Akgun EE Gonen ZB Local application of gingiva-derived mesenchymal stem cells on experimental periodontitis in rats. J Periodontol. (2024) 95(5):456–68. 10.1002/JPER.23-0246 37787060

[121] Li J Xu S-Q Zhang K Zhang W-J Liu H-L Xu Z Treatment of gingival defects with gingival mesenchymal stem cells derived from human fetal gingival tissue in a rat model. Stem Cell Res Ther. (2018) 9(1):27. 10.1186/s13287-017-0751-7 29402326 PMC5800013

[122] Qiu J Wang X Zhou H Zhang C Wang Y Huang J Enhancement of periodontal tissue regeneration by conditioned media from gingiva-derived or periodontal ligament-derived mesenchymal stem cells: a comparative study in rats. Stem Cell Res Ther. (2020) 11(1):42. 10.1186/s13287-019-1546-9 32014015 PMC6998241

[123] Xu Q-C Wang Z-G Ji Q-X Yu X-B Xu X-Y Yuan C-Q Systemically transplanted human gingiva-derived mesenchymal stem cells contributing to bone tissue regeneration. Int J Clin Exp Pathol. (2014) 7(8):4922. https://pmc.ncbi.nlm.nih.gov/articles/PMC4152053/25197363 PMC4152053

[124] Pimentel KF de Lima Sousa MG dos Santos Passos A Farias RJ Guerra JM Costa FWG The impact of partially removing the Bichat fat pad in the linear facial measurements, satisfaction with facial aesthetics and quality of life: a single-arm CONSORT-guided clinical trial. Clin Oral Investig. (2023) 27(1):249–62. 10.1007/s00784-022-04718-0 36152084

[125] Akita D Tsukimura N Kazama T Takahashi R Taniguchi Y Inoue J Regeneration of two-walled infrabony periodontal defects in swine after buccal fat pad-derived dedifferentiated fat cell autologous transplantation. Biomolecules. (2025) 15(4):604. 10.3390/biom15040604 40305349 PMC12024700

[126] Abdel-Fatah R Elkashty A El-Sharkawy H. The use of free buccal pad fat graft as a viable therapeutic modality in localized gingival recession: a randomized controlled clinical trial. BMC Oral Health. (2025) 25(1):780. 10.1186/s12903-025-06150-8 40413437 PMC12103013

[127] Mohammadi I Najafi A Razavi SM Khazaei S Tajmiri G. Effect of buccal fat autotransplantation on improving the alveolar socket bone regeneration: an *in-vivo* study. Heliyon. (2024) 10(6):e28131. 10.1016/j.heliyon.2024.e28131 38524537 PMC10958428

[128] Genova T Cavagnetto D Tasinato F Petrillo S Ruffinatti FA Mela L Isolation and characterization of buccal fat pad and dental pulp MSCs from the same donor. Biomedicines. (2021) 9(3):265. 10.3390/biomedicines9030265 33800030 PMC7999167

[129] Nazhvani FD Amirabad LM Azari A Namazi H Hosseinzadeh S Samanipour R Effects of *in vitro* low oxygen tension preconditioning of buccal fat pad stem cells on *in vivo* articular cartilage tissue repair. Life Sci. (2021) 280:119728. 10.1016/j.lfs.2021.119728 34144057

[130] Farré-Guasch E Martí-Pagès C Hernández-Alfaro F Klein-Nulend J Casals N. Buccal fat pad, an oral access source of human adipose stem cells with potential for osteochondral tissue engineering: an *in vitro* study. Tissue Eng Part C: Methods. (2010) 16(5):1083–94. 10.1089/ten.tec.2009.0487 20078198

[131] Shiraishi T Sumita Y Wakamastu Y Nagai K Asahina I. Formation of engineered bone with adipose stromal cells from buccal fat pad. J Dent Res. (2012) 91(6):592–7. 10.1177/0022034512445633 22538411

[132] Linero I Chaparro O. Paracrine effect of mesenchymal stem cells derived from human adipose tissue in bone regeneration. PLoS One. (2014) 9(9):e107001. 10.1371/journal.pone.0107001 25198551 PMC4157844

[133] Miura M Gronthos S Zhao M Lu B Fisher LW Robey PG SHED: stem cells from human exfoliated deciduous teeth. Proc Natl Acad Sci USA. (2003) 100(10):5807–12. 10.1073/pnas.0937635100 12716973 PMC156282

[134] Doğan A Demirci S Şahin F. *In vitro* differentiation of human tooth germ stem cells into endothelial-and epithelial-like cells. Cell Biol Int. (2015) 39(1):94–103. 10.1002/cbin.10357 25077982

[135] Nourbakhsh N Baniebrahimi G Talebi S Talebi A Esfahani MHN Movahedian B Subcutaneous implantation of tooth germ stem cells over the masseter muscle in mice: an *in vivo* pilot study. Regenerative Therapy. (2025) 28:536. 10.1016/j.reth.2025.01.021 40027990 PMC11869380

[136] Yalvac ME Ramazanoglu M Rizvanov AA Sahin F Bayrak OF Salli U Isolation and characterization of stem cells derived from human third molar tooth germs of young adults: implications in neo-vascularization, osteo-, adipo-and neurogenesis. Pharmacogenomics J. (2010) 10(2):105–13. 10.1038/tpj.2009.40 19721467

[137] Guzmán-Uribe D Estrada KNA Guillén A Pérez SM Ibáñez RR. Development of a three-dimensional tissue construct from dental human ectomesenchymal stem cells: *in vitro* and *in vivo* study. Open Dent J. (2012) 6:226. 10.2174/1874210601206010226 23308086 PMC3540401

[138] Gronthos S Mankani M Brahim J Robey PG. Postnatal human dental pulp stem cells (DPSCs) *in vitro* and *in vivo*. Proc Natl Acad Sci USA. (2000) 97(25):13625–30. 10.1073/pnas.240309797 11087820 PMC17626

[139] Seo B-M Miura M Gronthos S Bartold PM Batouli S Brahim J Investigation of multipotent postnatal stem cells from human periodontal ligament. Lancet. (2004) 364(9429):149–55. 10.1016/S0140-6736(04)16627-0 15246727

[140] Sonoyama W Liu Y Fang D Yamaza T Seo B-M Zhang C Mesenchymal stem cell-mediated functional tooth regeneration in swine. PLoS One. (2006) 1(1):e79. 10.1371/journal.pone.0000079 17183711 PMC1762318

[141] Zhang Q Shi S Liu Y Uyanne J Shi Y Shi S Mesenchymal stem cells derived from human gingiva are capable of immunomodulatory functions and ameliorate inflammation-related tissue destruction in experimental colitis. The Journal of Immunology. (2009) 183(12):7787–98. 10.4049/jimmunol.0902318 19923445 PMC2881945

[142] Ikeda E Yagi K Kojima M Yagyuu T Ohshima A Sobajima S Multipotent cells from the human third molar: feasibility of cell-based therapy for liver disease. Differentiation. (2008) 76(5):495–505. 10.1111/j.1432-0436.2007.00245.x 18093227

[143] Bi R Lyu P Song Y Li P Song D Cui C Function of dental follicle progenitor/stem cells and their potential in regenerative medicine: from mechanisms to applications. Biomolecules. (2021) 11(7):997. 10.3390/biom11070997 34356621 PMC8301812

[144] Kang J Fan W Deng Q He H Huang F. Stem cells from the apical papilla: a promising source for stem cell-based therapy. BioMed Res Int. (2019) 2019(1):6104738. 10.1155/2019/6104738 30834270 PMC6374798

[145] Sachdeva S Mani A Saluja H Chatterjee A. Ozonized hydrogels for clinical and domiciliary management in periodontal regenerative therapy an annotation. J Cell Biotechnol. (2023) 9(2):121–9. 10.3233/JCB-230101

[146] Tu C-C Cheng N-C Yu J Pan Y-X Tai W-C Chen Y-C Adipose-derived stem cell spheroid-laden microbial transglutaminase cross-linked gelatin hydrogel for treating diabetic periodontal wounds and craniofacial defects. Stem Cell Res Ther. (2023) 14(1):20. 10.1186/s13287-023-03238-2 36737813 PMC9898981

[147] Li G Li Z Li L Liu S Wu P Zhou M Stem cell-niche engineering via multifunctional hydrogel potentiates stem cell therapies for inflammatory bone loss. Adv Funct Mater. (2023) 33(2):2209466. 10.1002/adfm.202209466

[148] Roldan L Montoya C Solanki V Cai KQ Yang M Correa S A novel injectable piezoelectric hydrogel for periodontal disease treatment. ACS Appl Mater Interfaces. (2023) 15(37):43441–54. 10.1021/acsami.3c08336 37672788

[149] Mai Z Mai Y Huang X Ning S Liao H. Evaluation of anti-inflammatory and antibacterial properties of photo-thermal hydrogel as dual functional platform for management of periodontitis. Int J Nanomed. (2025) 20:2923–34. 10.2147/IJN.S508864 PMC11911236 40098724

[150] Tong X Xu Y Zhang T Deng C Xun J Sun D Exosomes from CD133+ human urine-derived stem cells combined adhesive hydrogel facilitate rotator cuff healing by mediating bone marrow mesenchymal stem cells. J Orthop Translat. (2023) 39:100–12. 10.1016/j.jot.2023.02.002 36879794 PMC9984782

[151] Ferjaoui Z López-Muñoz R Akbari S Chandad F Mantovani D Rouabhia M Design of alginate/gelatin hydrogels for biomedical applications: fine-tuning osteogenesis in dental pulp stem cells while preserving other cell behaviors. Biomedicines. (2024) 12(7):1510. 10.3390/biomedicines12071510 39062083 PMC11274465

[152] Samiei M Abdolahinia ED Fathi M Barar J Omidi Y. Chitosan-based bioactive hydrogels for osteogenic differentiation of dental pulp stem cells. J Drug Deliv Sci Technol. (2022) 73:103478. 10.1016/j.jddst.2022.103478

[153] Wang T Yan J Zhang S Qi N Zhang Y Li G Silk fibroin microspheres loaded Rehmannia Liuwei extract for the protection of endothelial cells from the inhibitory effects. Colloids Surf B Biointerfaces. (2024) 241:114034. 10.1016/j.colsurfb.2024.114034 38878662

[154] Han D Wang X Song S Shen H Lu Y Zheng Z Advances in multifunctional medical sutures for wound healing: a review. Coll Surf B Biointerfaces. (2025) 256:115062. 10.1016/j.colsurfb.2025.115062 40876245

[155] Liang M Zuo D Wang F Zhang B Tian J Huang X Drug-eluting vascular stents with surface modification of anticoagulation and pro-endothelialization. Biomacromolecules. (2025) 26(10):7074–84. 10.1021/acs.biomac.5c01382 40990391

[156] Yang J Yang F Xu W Yu X Zheng Z Wang X Composite hydrogel dressing with drug-release capability and enhanced mechanical performance. Biomacromolecules. (2025) 26(9):5715–26. 10.1021/acs.biomac.5c00505 40793969 PMC12421659

[157] Li Z Chen L Wu J Chen Y Zhu Y Li G A review of 3D bioprinting for organoids. Med Rev. (2025) 5(4):318–38. 10.1515/mr-2024-0089 PMC12362060 40838107

[158] Miao G Liang L Li W Ma C Pan Y Zhao H 3D Bioprinting of a bioactive composite scaffold for cell delivery in periodontal tissue regeneration. Biomolecules. (2023) 13(7):1062. 10.3390/biom13071062 37509098 PMC10377653

[159] Isik M Vargel I Ozgur E Cam SB Korkusuz P Emregul E Human periodontal ligament stem cells-derived exosomes-loaded hybrid hydrogel enhances the calvarial defect regeneration in middle-age rats. Mater Today Commun. (2023) 36:106869. 10.1016/j.mtcomm.2023.106869

[160] He T Yin Y Li X Zhu L Zheng Z Li G Carbonic anhydrase-integrated silk hydrogels for efficient microalgae growth and carbon fixation. ACS ES&T Eng. (2025) 5(6):1373–84. 10.1021/acsestengg.4c00831

[161] Wang X Shen H Shou D Liu Y Wang T Zheng Z A braided surgical silk suture with controllable biodegradability via enzymatic hydrolysis. Polym Degrad Stab. (2024) 230:111080. 10.1016/j.polymdegradstab.2024.111080

[162] Mei N Wu Y Chen B Zhuang T Yu X Sui B 3D-printed Mesoporous bioactive glass/GelMA biomimetic scaffolds for osteogenic/cementogenic differentiation of periodontal ligament cells. Front Bioeng Biotechnol. (2022) 10:950970. 10.3389/fbioe.2022.950970 36329698 PMC9623086

[163] Han P Raveendran N Liu C Basu S Jiao K Johnson N 3D Bioprinted small extracellular vesicles from periodontal cells enhance mesenchymal stromal cell function. Biomater Adv. (2024) 158:213770. 10.1016/j.bioadv.2024.213770 38242057

[164] Lin H-H Chao P-HG Tai W-C Chang P-C. 3D-printed collagen-based waveform microfibrous scaffold for periodontal ligament reconstruction. Int J Mol Sci. (2021) 22(14):7725. 10.3390/ijms22147725 34299345 PMC8307958

[165] Tian Y Liu M Liu Y Shi C Wang Y Liu T The performance of 3D bioscaffolding based on a human periodontal ligament stem cell printing technique. Journal of Biomed Mater Res Part A. (2021) 109(7):1209–19. 10.1002/jbm.a.37114 33021062

[166] Zhu Y Wang W Chen Q Ren T Yang J Li G Bioprinted PDLSCs with high-concentration GelMA hydrogels exhibit enhanced osteogenic differentiation *in vitro* and promote bone regeneration *in vivo*. Clin Oral Investig. (2023) 27(9):5153–70. 10.1007/s00784-023-05135-7 37428274

[167] Kandalam U Kawai T Ravindran G Brockman R Romero J Munro M Predifferentiated gingival stem cell-induced bone regeneration in rat alveolar bone defect model. Tissue Eng, Part A. (2021) 27(5-6):424–36. 10.1089/ten.tea.2020.0052 32729362 PMC8098763

[168] Yao J Zhang C Yu C Wu L Tan L Li W Immune microenvironment modulation for treating inflammatory periodontal bone defects using a dynamic dual-responsive hydrogel. J Mater Chem B. (2025) 13(26):7819–37. 10.1039/D5TB00784D 40478245

[169] Wang X-y Li Q-l Liu Z Wu Y-x Zhang Z-x Mao J Thermosensitive composite hydrogel with antibacterial, immunomodulatory, and osteogenic properties promotes periodontal bone regeneration via staged release of doxycycline and proanthocyanidin. Mater Today Chem. (2024) 38:102052. 10.1016/j.mtchem.2024.102052

[170] Yu Y You Z Li X Lou F Xiong D Ye L Injectable nanocomposite hydrogels with strong antibacterial, osteoinductive, and ROS-scavenging capabilities for periodontitis treatment. ACS Appl Mater Interfaces. (2024) 16(12):14421–33. 10.1021/acsami.3c16577 38497587

[171] Li Q Wang D Xiao C Wang H Dong S. Advances in hydrogels for periodontitis treatment. ACS Biomater Sci Eng. (2024) 10(5):2742–61. 10.1021/acsbiomaterials.4c00220 38639082

[172] Zhou J Liu Y. Research progress of hydrogel therapy to improve hypoxic environment of periodontal tissues and promote periodontal regeneration. J Oral Biol Craniofac Res. (2025) 15(4):869–79. 10.1016/j.jobcr.2025.06.005 40586108 PMC12205322

[173] Peng C Wang G Li J Wang Y Shu Z Tang M Ros-responsive and scavenging bifunctional hydrogel enables co-delivery of anti-inflammatory agent and osteogenetic nanoparticle for periodontitis treatment. Mater Des. (2024) 239:112777. 10.1016/j.matdes.2024.112777

[174] Pinho LC Ferreira M Graça A Marto J Colaço B Fernandes MH Tannic acid-enhanced gelatin-based composite hydrogel as a candidate for canine periodontal regeneration. Gels. (2025) 11(8):650. 10.3390/gels11080650 40868780 PMC12385206

[175] Sen S Sahu R Dua TK Paul P Nandi G. Advancements of multifunctional hydrogels in treating periodontal diseases: a concise review. Next Mater. (2025) 8:100825. 10.1016/j.nxmate.2025.100825

[176] Mamidi N De Silva FF Vacas AB Gutiérrez Gómez JA Montes Goo NY Mendoza DR Multifaceted hydrogel scaffolds: bridging the gap between biomedical needs and environmental sustainability. Adv Healthcare Mater. (2024) 13(27):2401195. 10.1002/adhm.202401195 38824416

[177] Lan X Wang Y Yin M. Enhancing periodontal ligament regeneration via PDLSC delivery using electrospun PCL/collagen/cellulose acetate scaffolds and collagen hydrogel incorporated with curcumin-loaded ZIF-8 nanoparticles. Int J Nanomed. (2025) 20:887–906. 10.2147/IJN.S492274 PMC11761539 39867310

[178] Ouyang Z Chen X Wang Z Xu Y Deng Z Xing L Azithromycin-loaded PLGA microspheres coated with silk fibroin ameliorate inflammation and promote periodontal tissue regeneration. Regen Biomater. (2025) 12:rbae146. 10.1093/rb/rbae146 39791015 PMC11717352

[179] Isaac A Jivan F Xin S Hardin J Luan X Pandya M Microporous bio-orthogonally annealed particle hydrogels for tissue engineering and regenerative medicine. ACS Biomater Sci Eng. (2019) 5(12):6395–404. 10.1021/acsbiomaterials.9b01205 33417792 PMC7992163

[180] Zhang G Zhang M Feng Q Wang R Mei H Xing K Supramolecular composite hydrogel loaded with CaF2 nanoparticles promotes the recovery of periodontitis bone resorption. ACS Appl Mater Interfaces. (2024) 16(35):45929–47. 10.1021/acsami.4c07210 39183483

[181] Liu Y Yan J Chen L Liao Y Huang L Tan J. Multifunctionalized and dual-crosslinked hydrogel promotes inflammation resolution and bone regeneration via nlrp3 inhibition in periodontitis. Small Struct. (2024) 5(3):2300281. 10.1002/sstr.202300281

[182] Guo Y Shao Z Wang W Liu H Zhao W Wang L Periodontium-mimicking, multifunctional biomass-based hydrogel promotes full-course socket healing. Biomacromolecules. (2024) 25(2):1246–61. 10.1021/acs.biomac.3c01221 38305191

[183] Ma Y Ji Y Zhong T Wan W Yang Q Li A Bioprinting-based PDLSC-ECM screening for *in vivo* repair of alveolar bone defect using cell-laden, injectable and photocrosslinkable hydrogels. ACS Biomater Sci Eng. (2017) 3(12):3534–45. 10.1021/acsbiomaterials.7b00601 33445388

[184] Luan Q Qiao R Wu X Shan J Song C Zhao X Plant-derived Chinese herbal hydrogel microneedle patches for wound healing. Small. (2024) 20(45):2404850. 10.1002/smll.202404850 39073298

[185] Song C Lu M Li N Gu H Li M Lu L MXene-integrated responsive hydrogel microneedles for oral ulcers healing. Smart Med. (2025) 4(1):e135. 10.1002/smmd.135 40059966 PMC11862566

[186] Guo H Huang S Yang X Wu J Kirk TB Xu J Injectable and self-healing hydrogels with double-dynamic bond tunable mechanical, gel–sol transition and drug delivery properties for promoting periodontium regeneration in periodontitis. ACS Appl Mater Interfaces. (2021) 13(51):61638–52. 10.1021/acsami.1c18701 34908393

[187] Edwards SD Hou S Brown JM Boudreau RD Lee Y Kim YJ Fast-curing injectable microporous hydrogel for *in situ* cell encapsulation. ACS Appl Bio Mater. (2022) 5(6):2786–94. 10.1021/acsabm.2c00214 35576622 PMC9290187

[188] Meng X Zhu Y Tan H Daraqel B Ming Y Li X The cytoskeleton dynamics-dependent LINC complex in periodontal ligament stem cells transmits mechanical stress to the nuclear envelope and promotes YAP nuclear translocation. Stem Cell Res Ther. (2024) 15(1):284. 10.1186/s13287-024-03884-0 39243052 PMC11380336

[189] Song C Liu R Fang Y Gu H Wang Y. Developing functional hydrogels for treatment of oral diseases. Smart Med. (2024) 3(3):e20240020. 10.1002/SMMD.20240020 39420948 PMC11425053

[190] He X-T Li X Xia Y Yin Y Wu R-X Sun H-H Building capacity for macrophage modulation and stem cell recruitment in high-stiffness hydrogels for complex periodontal regeneration: experimental studies *in vitro* and in rats. Acta Biomater. (2019) 88:162–80. 10.1016/j.actbio.2019.02.004 30735811

[191] Yin J Lei Q Luo X Jiang T Zou X Schneider A Degradable hydrogel fibers encapsulate and deliver metformin and periodontal ligament stem cells for dental and periodontal regeneration. J Appl Oral Sci. (2023) 31:e20220447. 10.1590/1678-7757-2022-0447 37132700 PMC10159044

[192] Chien K-H Chang Y-L Wang M-L Chuang J-H Yang Y-C Tai M-C Promoting induced pluripotent stem cell-driven biomineralization and periodontal regeneration in rats with maxillary-molar defects using injectable BMP-6 hydrogel. Sci Rep. (2018) 8(1):114. 10.1038/s41598-017-18415-6 29311578 PMC5758833

[193] Li M Tian J Yu K Liu H Yu X Wang N A ROS-responsive hydrogel incorporated with dental follicle stem cell-derived small extracellular vesicles promotes dental pulp repair by ameliorating oxidative stress. Bioact Mater. (2024) 36:524–40. 10.1016/j.bioactmat.2024.06.036 39072284 PMC11279300

[194] Xia Z Zhao B Xiang J Xu K Luo K Yu L-X Injectable pH-responsive hydrogel adapted to gingival crevicular fluid microenvironment for periodontitis therapy. ACS Appl Mater Interfaces. (2025) 17(21):31357–67. 10.1021/acsami.5c02776 40383914

[195] Lu X Hu S Zhang Z Bao J Cao B Xie J A pH-sensitive CuHP composite hydrogel featuring antibacterial, antioxidant, and osteogenic properties for treating diabetic periodontitis. Regen Biomater. (2025) 12:rbaf065. 10.1093/rb/rbaf065 40979829 PMC12448283

[196] Ugrinovic V Panic V Spasojevic P Seslija S Bozic B Petrovic R Strong and tough, pH sensible, interpenetrating network hydrogels based on gelatin and poly (methacrylic acid). Polym Eng Sci. (2022) 62(3):622–36. 10.1002/pen.25870

[197] Chen Y Guo B Zhang Y Bao X Li D Lin J. Injectable hypoxia-preconditioned human exfoliated deciduous teeth stem cells encapsulated within GelMA-AMP microspheres for bone regeneration in periodontitis. Coll Surf B Biointerfaces. (2025) 247:114452. 10.1016/j.colsurfb.2024.114452 39689590

[198] Amiri MA Amiri D Hamedani S. Thermosensitive hydrogels for periodontal regeneration: a systematic review of the evidence. Clin Exp Dent Res. (2024) 10(6):e70029. 10.1002/cre2.70029 39539029 PMC11561135

[199] Yang H Nguyen KT Leong DT Tan NS Tay CY. Soft material approach to induce oxidative stress in mesenchymal stem cells for functional tissue repair. ACS Appl Mater Interfaces. (2016) 8(40):26591–9. 10.1021/acsami.6b09222 27608498

[200] Song C Zhang X Lu M Zhao Y. Bee sting-inspired inflammation-responsive microneedles for periodontal disease treatment. Research. (2023) 6:0119. 10.34133/research.0119 37223473 PMC10202374

[201] Yuan P Luo Y Luo Y Ma L. A “sandwich” cell culture platform with NIR-responsive dynamic stiffness to modulate macrophage phenotypes. Biomater Sci. (2021) 9(7):2553–61. 10.1039/D0BM02194F 33576368

[202] Sousa AB Barbosa JN. The use of specialized pro-resolving mediators in biomaterial-based immunomodulation. J Funct Biomater. (2023) 14(4):223. 10.3390/jfb14040223 37103313 PMC10145769

[203] Morsy BM El Domiaty S Meheissen MAM Heikal LA Meheissen MA Aly NM. Omega-3 nanoemulgel in prevention of radiation-induced oral mucositis and its associated effect on microbiome: a randomized clinical trial. BMC Oral Health. (2023) 23(1):612. 10.1186/s12903-023-03276-5 37648997 PMC10470147

[204] Ge X Hu J Qi X Shi Y Chen X Xiang Y An immunomodulatory hydrogel featuring antibacterial and reactive oxygen species scavenging properties for treating periodontitis in diabetes. Adv Mater. (2025) 37(3):2412240. 10.1002/adma.202412240 39610168

[205] Kharaziha M Baidya A Annabi N. Rational design of immunomodulatory hydrogels for chronic wound healing. Adv Mater. (2021) 33(39):2100176. 10.1002/adma.202100176 PMC8489436 34251690

[206] Banerjee A Singh P Sheikh PA Kumar A Koul V Bhattacharyya J. Simultaneous regulation of AGE/RAGE signaling and MMP-9 expression by an immunomodulating hydrogel accelerates healing in diabetic wounds. Biomater Adv. (2024) 163:213937. 10.1016/j.bioadv.2024.213937 38968788

[207] Huang X Zhang Y Huang Y Hu Q Mai Z Qin Y Protease-Responsive protein hydrogel enabling spatiotemporal ClO2 release for precision treatment of intracellular infections and periodontitis. Mater Des. (2025) 256:114321. 10.1016/j.matdes.2025.114321

[208] Xie C Zhang Q. H_2_S-Scavenging hydrogel alleviating mitochondria damage to control periodontitis. J Dent Res. (2025) 104(2):172–82. 10.1177/00220345241291540 39629939

[209] Shen S Liu R Song C Shen T Zhou Y Guo J Fish scale-derived scaffolds with MSCs loading for photothermal therapy of bone defect. Nano Res. (2023) 16(5):7383–92. 10.1007/s12274-023-5460-1

[210] Song C Wu X Wang Y Wang J Zhao Y. Cuttlefish-inspired photo-responsive antibacterial microparticles with natural melanin nanoparticles spray. Small. (2024) 20(19):2310444. 10.1002/smll.202310444 38050927

[211] Song C Huang D Zhao C Zhao Y. Abalone-inspired adhesive and photo-responsive microparticle delivery systems for periodontal drug therapy. Adv Sci. (2022) 9(30):2202829. 10.1002/advs.202202829 PMC9596845 36041051

[212] Fu Y Wang Y Cheng B Zou R Wan W. Substrate stiffness regulates the osteogenesis of PDLSCs via ERK-mediated YAP nuclear translocation. Int Dent J. (2025) 75(6):103852. 10.1016/j.identj.2025.103852 41075461 PMC12547731

[213] Lee S Choi S Kwon H Kim E Lee E Kim SM Spatiotemporal control of autonomous adipogenesis of pre-adipocyte spheroids by bioactive nanofibers and soft hydrogel microenvironments. Biomater Sci. (2025) 13(18):5096–110. 10.1039/D5BM00901D 40747964

[214] Assadi Z Rezvanian P Gounani Z Ejeian F Zarrabi A Masaeli E. Multilayered nanocomposite membrane orchestrating targeted dual release strategies for enhanced guided bone regeneration. Chem Eng J. (2024) 484:149237. 10.1016/j.cej.2024.149237

[215] Qiu S Xu X Feng Z Li L Wang X Zheng K Bioactive glass-laden ionically crosslinked pectin microspheres with pro-osteogenic and antibacterial activities for periodontal regeneration. Ceram Int. (2025) 51:35323–36. 10.1016/j.ceramint.2025.05.253

[216] Wu S Chai Z Yang Y Ding R Gao B Chen C Effect of matrix stiffness on the osteogenic differentiation of human periodontal ligament stem cells in a three-dimensional culture hydrogel: a preliminary study. ACS Biomater Sci Eng. (2025) 11(9):5616–26. 10.1021/acsbiomaterials.5c01151 40856628

[217] Liu J Li P Chen Y Shi Y Chen K Liu J Pre-vascularized hydrogel co-encapsulating SHEDs and HUVECs for dental pulp regeneration. Biomater Adv. (2025) 180:214539. 10.1016/j.bioadv.2025.214539 41061320

[218] Kwon H Lee S Byun H Huh SJ Lee E Kim E Engineering pre-vascularized 3D tissue and rapid vascular integration with host blood vessels via co-cultured spheroids-laden hydrogel. Biofabrication. (2024) 16(2):025029. 10.1088/1758-5090/ad30c6 38447223

[219] Dong S Mei Y Zhang Y Bu W Zhang Y Sun C A novel therapeutic calcium peroxide loaded injectable bio-adhesive hydrogel against periodontitis. Int Dent J. (2025) 75(1):352–62. 10.1016/j.identj.2024.05.013 39127517 PMC11806305

[220] He T Tan Q Huang Y Chen J Tan J Zhou C Extracellular adipose matrix hydrogel laden with adipose-derived stem cell modulates macrophage polarization for enhanced full-thickness skin wound repair. Biomacromolecules. (2025) 26(6):3588–604. 10.1021/acs.biomac.5c00194 40340431

[221] Lian S Mu Z Yuan Z Shafiq M Mo X Mu W. Methacrylated gelatin and platelet-rich plasma based hydrogels promote regeneration of critical-sized bone defects. Regen Biomater. (2024) 11:rbae022. 10.1093/rb/rbae022 38567105 PMC10985677

[222] An N Yan X Qiu Q Zhang Z Zhang X Zheng B Human periodontal ligament stem cell sheets activated by graphene oxide quantum dots repair periodontal bone defects by promoting mitochondrial dynamics dependent osteogenic differentiation. J Nanobiotechnol. (2024) 22(1):133. 10.1186/s12951-024-02422-7 PMC10976692 38539195

[223] Yu J-L Yang C Liu L Lin A Guo S-J Tian W-D. Optimal good manufacturing practice-compliant production of dental follicle stem cell sheet and its application in sprague-dawley rat periodontitis. World J Stem Cells. (2025) 17(5):104116. 10.4252/wjsc.v17.i5.104116 40503360 PMC12149792

[224] Sowmya S Mony U Jayachandran P Reshma S Kumar RA Arzate H Tri-layered nanocomposite hydrogel scaffold for the concurrent regeneration of cementum, periodontal ligament, and alveolar bone. Adv Healthcare Mater. (2017) 6(7):1601251. 10.1002/adhm.201601251 28128898

[225] Yang X Ma Y Wang X Yuan S Huo F Yi G A 3D-bioprinted functional module based on decellularized extracellular matrix bioink for periodontal regeneration. Adv Sci. (2023) 10(5):2205041. 10.1002/advs.202205041 PMC9929114 36516309

[226] Wang J Yang F Chen R Yang X Wang J Zhang H. Hydrogel composite incorporating deferoxamine-loaded gelatin-based microspheres enhance angiogenesis ability of dental pulp stem cells. ACS Omega. (2025) 10(12):12579–89. 10.1021/acsomega.5c00445 40191326 PMC11966253

[227] Divband B Aghazadeh M Al-Qaim ZH Samiei M Hussein FH Shaabani A Bioactive chitosan biguanidine-based injectable hydrogels as a novel BMP-2 and VEGF carrier for osteogenesis of dental pulp stem cells. Carbohydr Polym. (2021) 273:118589. 10.1016/j.carbpol.2021.118589 34560990

[228] Wang L Wei X He X Xiao S Shi Q Chen P Osteoinductive dental pulp stem cell-derived extracellular vesicle-loaded multifunctional hydrogel for bone regeneration. ACS Nano. (2024) 18(12):8777–97. 10.1021/acsnano.3c11542 38488479

[229] Yamada S Al-Sharabi N Torelli F Volponi AA Sandven L Ueda M Harnessing the antioxidative potential of dental pulp stem cell-conditioned medium in photopolymerized GelMA hydrogels. Biomater Res. (2024) 28:0084. 10.34133/bmr.0084 39290361 PMC11406670

[230] Chen Y Zhang F Fu Q Liu Y Wang Z Qi N. *In vitro* proliferation and osteogenic differentiation of human dental pulp stem cells in injectable thermo-sensitive chitosan/β-glycerophosphate/hydroxyapatite hydrogel. J Biomater Appl. (2016) 31(3):317–27. 10.1177/0885328216661566 27496540

[231] Moshaverinia A Chen C Akiyama K Ansari S Xu X Chee WW Alginate hydrogel as a promising scaffold for dental-derived stem cells: an *in vitro* study. J Mater Sci: Mater Med. (2012) 23(12):3041–51. 10.1007/s10856-012-4759-3 22945383

[232] Fawzy El-Sayed KM Paris S Becker ST Neuschl M De Buhr W Sälzer S Periodontal regeneration employing gingival margin-derived stem/progenitor cells: an animal study. J Clin Periodontol. (2012) 39(9):861–70. 10.1111/j.1600-051X.2012.01904.x 22694281

[233] Ansari S Pouraghaei Sevari S Chen C Sarrion P Moshaverinia A. RGD-modified alginate–GelMA hydrogel sheet containing gingival mesenchymal stem cells: a unique platform for wound healing and soft tissue regeneration. ACS Biomater Sci Eng. (2021) 7(8):3774–82. 10.1021/acsbiomaterials.0c01571 34082525

[234] Moshaverinia A Chen C Xu X Akiyama K Ansari S Zadeh HH Bone regeneration potential of stem cells derived from periodontal ligament or gingival tissue sources encapsulated in RGD-modified alginate scaffold. Tissue Eng, Part A. (2014) 20(3-4):611–21. 10.1089/ten.tea.2013.0229 24070211 PMC3926152

[235] Pouraghaei Sevari S Kim JK Chen C Nasajpour A Wang C-Y Krebsbach PH Whitlockite-enabled hydrogel for craniofacial bone regeneration. ACS Appl Mater Interfaces. (2021) 13(30):35342–55. 10.1021/acsami.1c07453 34297530

[236] Deng J Pan J Han X Yu L Chen J Zhang W PDGFBB-modified stem cells from apical papilla and thermosensitive hydrogel scaffolds induced bone regeneration. Chem-Biol Interact. (2020) 316:108931. 10.1016/j.cbi.2019.108931 31874163

[237] Dutta SD Bin J Ganguly K Patel DK Lim K-T. Electromagnetic field-assisted cell-laden 3D printed poloxamer-407 hydrogel for enhanced osteogenesis. RSC Adv. (2021) 11(33):20342–54. 10.1039/D1RA01143J 35479929 PMC9033958

[238] Qu L Dubey N Ribeiro JS Bordini EAF Ferreira JA Xu J Metformin-loaded nanospheres-laden photocrosslinkable gelatin hydrogel for bone tissue engineering. J Mech Behav Biomed Mater. (2021) 116:104293. 10.1016/j.jmbbm.2020.104293 33588247 PMC8275125

[239] Ma Y Ji Y Huang G Ling K Zhang X Xu F. Bioprinting 3D cell-laden hydrogel microarray for screening human periodontal ligament stem cell response to extracellular matrix. Biofabrication. (2015) 7(4):044105. 10.1088/1758-5090/7/4/044105 26696269

[240] Ivanov AA Kuznetsova AV Popova OP Danilova TI Latyshev AV Yanushevich OO. Influence of extracellular matrix components on the differentiation of periodontal ligament stem cells in collagen I hydrogel. Cells. (2023) 12(19):2335. 10.3390/cells12192335 37830549 PMC10571948

[241] Saputra G Nugraha AP Budhy TI Rosari FS Lestari NAI Sari AA Nanohydroxyapatite-chitosan hydrogel scaffold with platelet rich fibrin and buccal fat pad derived stem cell for aggressive periodontitis treatment: a narrative review. Res J Pharm Technol. (2022) 15(12):5903–8. 10.52711/0974-360X.2022.00995

[242] Bastami F Paknejad Z Jafari M Salehi M Rad MR Khojasteh A. Fabrication of a three-dimensional β-tricalcium-phosphate/gelatin containing chitosan-based nanoparticles for sustained release of bone morphogenetic protein-2: implication for bone tissue engineering. Mater Sci Eng C. (2017) 72:481–91. 10.1016/j.msec.2016.10.084 28024612

[243] Ryu HS Abueva C Padalhin A Park SY Yoo SH Seo HH Oral ulcer treatment using human tonsil-derived mesenchymal stem cells encapsulated in trimethyl chitosan hydrogel: an animal model study. Stem Cell Res Ther. (2024) 15(1):103. 10.1186/s13287-024-03694-4 38589946 PMC11003084

[244] Arpornmaeklong P Boonyuen S Apinyauppatham K Pripatnanont P. Effects of oral cavity stem cell sources and serum-free cell culture on hydrogel encapsulation of mesenchymal stem cells for bone regeneration: an *in vitro* investigation. Bioengineering. (2024) 11(1):59. 10.3390/bioengineering11010059 38247936 PMC10812978

[245] Qu X Xie Z Zhang J Huang Y Zhao R Li N Regulating mitochondrial aging via targeting the gut-bone axis in BMSCs with oral hydrogel microspheres to inhibit bone loss. Small. (2025) 21(4):2409936. 10.1002/smll.202409936 39629509

[246] Da Silva D Crous A Abrahamse H. Enhancing osteoblast differentiation from adipose-derived stem cells using hydrogels and photobiomodulation: overcoming *in vitro* limitations for osteoporosis treatment. Curr Issues Mol Biol. (2024) 46(7):6346–65. 10.3390/cimb46070379 39057021 PMC11276038

[247] He X Chu X-Y Chen X Xiang Y-L Li Z-L Gao C-Y Dental pulp stem cell-derived extracellular vesicles loaded with hydrogels promote osteogenesis in rats with alveolar bone defects. Mol Med Rep. (2024) 31(1):29. 10.3892/mmr.2024.13393 39540371 PMC11582518

[248] El-Qashty R Youssef J Hany E. The role of erythropoietin-loaded hydrogel versus adipose derived stem cell secretome in the regeneration of tongue defects. BMC Oral Health. (2024) 24(1):1109. 10.1186/s12903-024-04835-0 39294639 PMC11411902

[249] Hua W Xiang J Wu Y Yang W Zhao L. Growth factor-encapsulated triphasic scaffolds of electrospun polylactic acid–polycaprolactone (PLA–PCL) nanofibrous mats combined with a directionally freeze-dried chitosan hydrogel for periodontal tissue regeneration. Mater Adv. (2023) 4(20):4798–811. 10.1039/D3MA00465A

[250] Qiao X Tang J Dou L Yang S Sun Y Mao H Dental pulp stem cell-derived exosomes regulate anti-inflammatory and osteogenesis in periodontal ligament stem cells and promote the repair of experimental periodontitis in rats. Int J Nanomed. (2023) 18:4683–703. 10.2147/IJN.S420967 PMC10441659 37608819

[251] Dong Z Lin Y Xu S Chang L Zhao X Mei X NIR-triggered tea polyphenol-modified gold nanoparticles-loaded hydrogel treats periodontitis by inhibiting bacteria and inducing bone regeneration. Mater Des. (2023) 225:111487. 10.1016/j.matdes.2022.111487

[252] Venkataiah VS Mehta D Fareed M Karobari MI. Advancements in osteoblast sourcing, isolation, and characterization for dental tissue regeneration: a review. Biomed Eng Online. (2025) 24(1):31. 10.1186/s12938-025-01363-y 40057736 PMC11890725

[253] Hodjat M Farshad F Gholami M Abdollahi M Saadat KASM. Histone deacetylase inhibitors restore the odontogenic differentiation potential of dental pulp stem cells under hyperglycemic conditions. Curr Stem Cell Res Ther. (2025) 20(4):441–8. 10.2174/011574888X309466240429051314 38712370

[254] Liu Y Liu Y Hu J Han J Song L Liu X Impact of allogeneic dental pulp stem cell injection on tissue regeneration in periodontitis: a multicenter randomized clinical trial. Signal Transduct Target Ther. (2025) 10(1):239. 10.1038/s41392-025-02320-w 40739139 PMC12311062

[255] Cao Y Liu Z Xie Y Hu J Wang H Fan Z Correction: adenovirus-mediated transfer of hepatocyte growth factor gene to human dental pulp stem cells under good manufacturing practice improves their potential for periodontal regeneration in swine. Stem Cell Res Ther. (2025) 16(1):73. 10.1186/s13287-025-04214-8 39980047 PMC11844060

[256] Tang W Huo F Ju R Gao X He M Long J Melatonin-coated nanofiber cell sheets promote periodontal regeneration through ROS scavenging and preservation of stemness. Chem Eng J. (2024) 497:154626. 10.1016/j.cej.2024.154626

[257] Patel MJ Shah KM Pillai JP. Awareness on dental pulp stem cells and their application in regenerative dentistry among dental and biotechnology professionals-an evaluative study. Indian J Dent Res. (2025) 36(2):139–43. 10.4103/ijdr.ijdr_422_23 40657978

[258] Alrehaili AA. Exploring parental knowledge, attitudes, and factors influencing decision-making in stem cell banking: rising the future of medical treatment. Cureus. (2024) 16(4):e58384. 10.7759/cureus.58384 38628380 PMC11020598

[259] Zeitlin BD. Banking on teeth–stem cells and the dental office. Biomed J. (2020) 43(2):124–33. 10.1016/j.bj.2020.02.003 32381462 PMC7283549

[260] Martins F Ribeiro MHL. Quality and regulatory requirements for the manufacture of master cell banks of clinical grade iPSCs: the EU and USA perspectives. Stem Cell Rev Rep. (2025) 21(3):645–79. 10.1007/s12015-024-10838-9 39821060

[261] Park G Rim YA Sohn Y Nam Y Ju JH. Replacing animal testing with stem cell-organoids: advantages and limitations. Stem Cell Rev Rep. (2024) 20(6):1375–86. 10.1007/s12015-024-10723-5 38639829 PMC11319430

[262] Vo QD Saito Y Ida T Nakamura K Yuasa S. The use of artificial intelligence in induced pluripotent stem cell-based technology over 10-year period: a systematic scoping review. PLoS One. (2024) 19(5):e0302537. 10.1371/journal.pone.0302537 38771829 PMC11108174

[263] Xia Y Cheng T Zhang C Zhou M Hu Z Kang F Human bone marrow mesenchymal stem cell-derived extracellular vesicles restore Th17/Treg homeostasis in periodontitis via miR-1246. FASEB J. (2023) 37(11):e23226. 10.1096/fj.202300674RR 37815505

[264] Yang J Gu H Zhu Y Shao J Chang H Zhou M Self-cascade ROS-trapping bioreaction system reverses stem cell oxidative stress fate for osteogenesis. Nano Today. (2024) 59:102514. 10.1016/j.nantod.2024.102514

[265] Yu N Rakian A Dean A Van Dyke TE. Specialized proresolving mediators facilitate the immunomodulation of the periodontal ligament stem cells. Front Dent Med. (2021) 2:701197. 10.3389/fdmed.2021.701197

[266] Zhao D-Z Yang R-L Wei H-X Yang K Yang Y-B Wang N-X Advances in the research of immunomodulatory mechanism of mesenchymal stromal/stem cells on periodontal tissue regeneration. Front Immunol. (2025) 15:1449411. 10.3389/fimmu.2024.1449411 39830512 PMC11739081

[267] Zhang Y Qin X Yang J Rogers HM Baban B Tian S. Clinical effect of immunomodulatory therapy in periodontitis: a systematic review and meta-analysis. Front Bioeng Biotechnol. (2025) 13:1693365. 10.3389/fbioe.2025.1693365 41356100 PMC12675453

[268] Tang Y Qi Y Chen Y Wang Y-Q Zhang C Sun Y Erythrocyte-mimicking nanovesicle targeting Porphyromonas gingivalis for periodontitis. ACS Nano. (2024) 18(32):21077–90. 10.1021/acsnano.4c02316 39088785

[269] Li X Zhang Z Xie J Cao B Wang X Yu Y A smart injectable hydrogel with dual responsivity to arginine gingipain A and reactive oxygen Species for multifunctional therapy of periodontitis. Small. (2025) 21(23):2408034. 10.1002/smll.202408034 40272094

[270] Yan J Chen H Pan Y Yan Y Tang S Zhou Q Magnetic labeling of physically tunable hydrogel-induced mesenchymal stem cell spheroids with IONPs for MRI tracking and bone regeneration. Nano Today. (2025) 61:102620. 10.1016/j.nantod.2024.102620

[271] Ma S Yan Q Li L Ni Y Chen K Xu B Photosensitive small intestinal submucosal hydrogels loaded with the KR-12-a5 peptide promote periodontal osteogenesis and antimicrobial activity. J Nanobiotechnol. (2025) 23(1):637. 10.1186/s12951-025-03601-w PMC12514852 41074027

[272] Pańczyszyn E Jaśko M Miłek O Niedziela M Męcik-Kronenberg T Hoang-Bujnowicz A Gellan gum hydrogels cross-linked with carbodiimide stimulates vacuolation of human tooth-derived stem cells *in vitro*. Toxicol in Vitro. (2021) 73:105111. 10.1016/j.tiv.2021.105111 33588021

[273] Anitua E Zalduendo M Troya M Erezuma I Lukin I Hernaez-Moya R Composite alginate-gelatin hydrogels incorporating PRGF enhance human dental pulp cell adhesion, chemotaxis and proliferation. Int J Pharm. (2022) 617:121631. 10.1016/j.ijpharm.2022.121631 35247496

[274] Cai G Ren L Yu J Jiang S Liu G Wu S A microenvironment-responsive, controlled release hydrogel delivering embelin to promote bone repair of periodontitis via anti-infection and osteo-immune modulation. Adv Sci. (2024) 11(34):2403786. 10.1002/advs.202403786 PMC11425865 38978324

[275] Lee J Jr Ng H-Y Lin Y-H Lin T-J Kao C-T Shie M-Y. The synergistic effect of cyclic tensile force and periodontal ligament cell-laden calcium silicate/gelatin methacrylate auxetic hydrogel scaffolds for bone regeneration. Cells. (2022) 11(13):2069. 10.3390/cells11132069 35805154 PMC9265804

[276] Han Z Bao L Yu Y Zhao Y Wang M Sun Y Injectable short-fiber hydrogel with fatigue resistance and antibacterial properties for synergistic periodontitis therapy. Chem Eng J. (2025) 520:166298. 10.1016/j.cej.2025.166298

[277] Lin Y Wang C Li S Xu S Jia B Zhang H Therapeutic effects of an injectable multifunctional thermosensitive hydrogel loaded with ascorbic acid carbon quantum dots and chitosan/chondroitin sulfate complex on periodontitis. Carbon N Y. (2025) 243:120570. 10.1016/j.carbon.2025.120570

[278] Liu S Wang Z Li Y Pan Z Huang L Cui J Erythropoietin-stimulated macrophage-derived extracellular vesicles in chitosan hydrogel rescue BMSCs fate by targeting EGFR to alleviate inflammatory bone loss in periodontitis. Advanced Science. (2025) 12(23):2500554. 10.1002/advs.202500554 40289904 PMC12199399

[279] Colangelo MT Guizzardi S Laschera L Meleti M Galli C. The effects of polynucleotides-based biomimetic hydrogels in tissue repair: a 2D and 3D *in vitro* study. Regen Med. (2025) 20(9):365–73. 10.1080/17460751.2025.2567177 41021673 PMC12502828

[280] Ding Z Yan Z Yuan X Tian G Wu J Fu L Apoptotic extracellular vesicles derived from hypoxia-preconditioned mesenchymal stem cells within a modified gelatine hydrogel promote osteochondral regeneration by enhancing stem cell activity and regulating immunity. J Nanobiotechnol. (2024) 22(1):74. 10.1186/s12951-024-02333-7 PMC10885680 38395929

[281] Zhang P Liu Z Pei H Ahmed A Wei Y Huang D. From passive to active: next-generation mechanically active dressings for wound healing. Acta Biomater. (2025) 208:168–89. 10.1016/j.actbio.2025.11.010 41207598

[282] Ding W Zhang T Fu R Yu C Zhang C Wang K Nano-sized coordination polymer particles (CPPs) for synergetic application in antibacterial and anticancer therapeutics. Chem Eng J. (2025) 516:163905. 10.1016/j.cej.2025.163905

[283] Zhang P Qi J Zhang R Zhao Y Yan J Gong Y Recent advances in composite hydrogels: synthesis, classification, and application in the treatment of bone defects. Biomater Sci. (2024) 12(2):308–29. 10.1039/D3BM01795H 38108454

[284] Liu Z Zhang P Pei H Wang Z Li L Ma X Enhance electrohydrodynamic direct-writing potential in bone tissue engineering: design innovations, multidisciplinary insight, and future direction. Adv Funct Mater. (2025):e19074. 10.1002/adfm.202519074

